# Transfer learning for identifying rainwater harvesting sites in training data-scarce catchments

**DOI:** 10.1038/s41598-026-51218-2

**Published:** 2026-05-05

**Authors:** Sri Priyanka Kommula, Raghvendra Singh, Bharat Lohani, Dongryeol Ryu, Stephan Winter

**Affiliations:** 1 Department of Civil Engineering, Indian Institute of Technology Kanpur, Kanpur, Uttar Pradesh India; 2 Department of Infrastructure Engineering, The University of Melbourne, Parkville, Victoria Australia

**Keywords:** Rainwater harvesting, Transfer learning, Machine learning, Geomorphic similarity, KL divergence, Suitability mapping, Data-scarce catchments, GR4J, Geospatial analysis, Environmental sciences, Hydrology, Natural hazards

## Abstract

Machine learning (ML) based approaches for rainwater harvesting (RWH) site suitability mapping often face limited data for training ML models. This study investigates whether the knowledge learned from data-rich catchments can be effectively transferred to the hydrologically and geomorphically similar and distinct basins. The transfer-learning (TL) framework is tested across three Indian catchments of different climatic conditions in the states of – Odisha, Maharashtra, and Tamil Nadu–using seven key influential predictors derived from LiDAR DEMs, geological and soil maps, and GR4J-simulated annual streamflow. A total of 18 experimental cases are designed, viz., intra, direct transfer, adaptation-based transfer, and multi-catchment combinations. Feature similarity between the source and target catchments is quantified using Kullback–Leibler (KL) divergence to systematically assess conditions for transfer feasibility. Four ML models–Support Vector Machine (SVM), Random Forest (RF), XGBoost (XGB), and KNN (K-Nearest Neighbour)–are trained over 100 randomized iterations using curated suitable and unsuitable RWH locations. Results show that performance of direct transfer declines sharply when dominant catchment features exhibit high divergence. RF and XGB are more resilient to cross-domain variability, while SVM and KNN are highly sensitive to feature mismatches. Incorporating a 20% target-domain sample substantially improves kappa and F1 scores by 25–40% in Odisha, 20–30% in Maharashtra, and 35–45% in Tamil Nadu, respectively, and stabilizes feature importance between the source and target domains. Multi-source training, which aggregates data from all three catchments, achieves the highest accuracy ($>$0.95) and kappa ($>$0.85). KL divergence analysis confirms that transferability depends on geomorphic similarity; that is, lower divergence enables effective transfer, whereas higher divergence reduces generalization. Overall, this study demonstrates that TL is feasible and beneficial for RWH site mapping, provides a practical and transferable tool for sustainable RWH planning in data-limited regions and also builds foundation for developing wide area scalable models by continuously updating the weights of influential thematic layers as new training data becomes available.

## Introduction

Rainwater harvesting (RWH) is widely recognized as a sustainable approach to addressing the growing demand for water, particularly in regions that depend heavily on monsoon rainfall and groundwater. The effectiveness of RWH structures largely depends on accurate site selection, as poorly chosen locations can result in ineffective water capture, waterlogging, or structural failure^1,2^, ultimately leading to resource wastage. While field-based surveys are effective for identifying suitable sites in small catchments, their application to larger regions is often limited by time, cost, and logistical constraints^3^. Traditionally, RWH site suitability analyses have relied on topographic and hydrological assessments that integrate key factors such as rainfall, slope, drainage density, soil, runoff, and land use/land cover (LULC)^4^.

To integrate these diverse criteria within a GIS environment, several multi-criteria analysis (MCA) approaches have been applied, such as Weighted Linear Combination^3,5^, the weighted overlay technique^6^ and Analytical Hierarchy Process (AHP)^7–9^. These methods collectively evaluate the potential of an area to support RWH structures but depend heavily on expert-assigned weights, introducing subjectivity that may result in inconsistent suitability outcomes across different studies.

With recent advances in machine learning (ML), data-driven approaches have emerged as powerful alternatives for spatial prediction and decision support in water resource management. Both deep learning and ML models–such as Random Forest (RF), Support Vector Machine (SVM), Convolutional Neural Network (CNN), and Recurrent Neural Network (RNN)–have been successfully applied for environmental mapping, hydrological prediction, and RWH site identification^4,10^. In addition, ML techniques have been widely adopted for diverse water resources applications, including streamflow and flood forecasting, groundwater level prediction, water quality modelling, and water supply–demand assessment^11–14^. These approaches enable the integration of large and heterogeneous datasets, improving predictive accuracy and supporting informed decision-making in complex hydrological systems. However, these approaches offer limited utility in regions with sparse or unevenly distributed training data, where model generalization deteriorates due to data scarcity and spatial heterogeneity^15^.

To overcome these challenges, transfer learning (TL) has been proposed as an alternative framework that enables the transfer or adaptation of knowledge learned from data-rich regions (source domains) to data-scarce regions (target domains)^16–18^. This approach enhances model generalization while minimizing the need for extensive field data collection, offering a scalable solution for RWH site identification in data-deficient environments.

In geospatial applications, TL has been effectively employed for various environmental and hazard prediction tasks such as landslide susceptibility mapping^17,18^, debris flow prediction^19^, flood depth estimation^16^, and streamflow modeling^20^. Studies have shown that when the source and target regions share similar physical and geomorphological characteristics, the performance of TL models is considerably better. For instance, Singh et al.^17^ used an ensemble transfer learning framework to reduce prediction errors in landslide susceptibility mapping for data-scarce Himalayan catchments. They quantified source–target similarity using Kullback–Leibler (KL) divergence, demonstrating that regions with lower divergence yielded superior transfer performance. Similarly, Wang et al.^18^ applied case-based reasoning and domain adaptation to select optimal source domains, achieving accuracies comparable to locally trained models.

Despite these advancements, the application of TL for RWH site suitability mapping remains largely unexplored. Most existing RWH studies rely on locally trained ML models or MCA approaches, both of which require extensive ground-truth data and field validation. In particular, ML-based approaches rely on the availability of already existing RWH locations to serve as training data. However, such data are limited to a few regions with prior water management interventions and documented RWH structures, and are often unavailable in new or under-explored regions due to sparse monitoring networks, limited infrastructure where water conservation planning is most needed. At the same time, many data-scarce catchments across India urgently require scientific support for RWH planning. This limitation restricts the direct application of conventional ML models and constrains their broader applicability.

To address this gap, the present study proposes a TL-based framework that enables knowledge transfer from data-rich to data-scarce catchments, thereby reducing the dependence on extensive local training data. The key contributions of this study include the development of a multi-catchment TL framework, the use of KL divergence to quantify source–target similarity, and a systematic evaluation of multiple ML models under different transfer learning configurations. Specifically, this study investigates whether knowledge from data-rich basins can be effectively transferred to hydro-geomorphically similar or distinct basins, specifically: (i) Can ML models trained in data-rich basins generalize to similar or distinct target basins? (ii) Does incorporating a small fraction of target-domain samples improve cross-domain predictions? (iii) Do multi-source models, trained on combined catchment data, enhance generalizability across heterogeneous regions?

Building on these insights, this study develops a TL framework for site suitability mapping for RWH structures such as loose boulder check dams and gabion dams, which typically range in width from 5 m to 30 m, across three hydro-geomorphically distinct catchments in India – Odisha, Maharashtra, and Tamil Nadu. The framework integrates ground-verified RWH sites, Light Detection and Ranging (LiDAR) derived terrain data, and hydrologically simulated streamflow from the GR4J model to assess model transferability under varying catchment conditions. To systematically assess the feasibility and performance of TL in RWH site identification, a set of controlled experimental cases was designed across the three catchments. Source–target similarity was quantified using the KL divergence to objectively assess domain alignment. Four machine learning models – SVM, RF, Extreme Gradient Boosting (XGB), and K-Nearest Neighbor (KNN) – were applied to examine model robustness and generalization across catchments.

The specific aims of this study are to (i) investigate how catchment similarity influences TL performance, (ii) determine the most effective TL configuration for improving prediction accuracy in data-scarce regions, and (iii) validate the overall feasibility of TL for scalable RWH site identification in heterogeneous Indian terrains. The proposed framework is expected to support practitioners and government agencies, such as the Ministry of Jal Shakti and the Central Water Commission, in planning and prioritizing water conservation structures across India.

## Study area and data

**Fig. 1:**
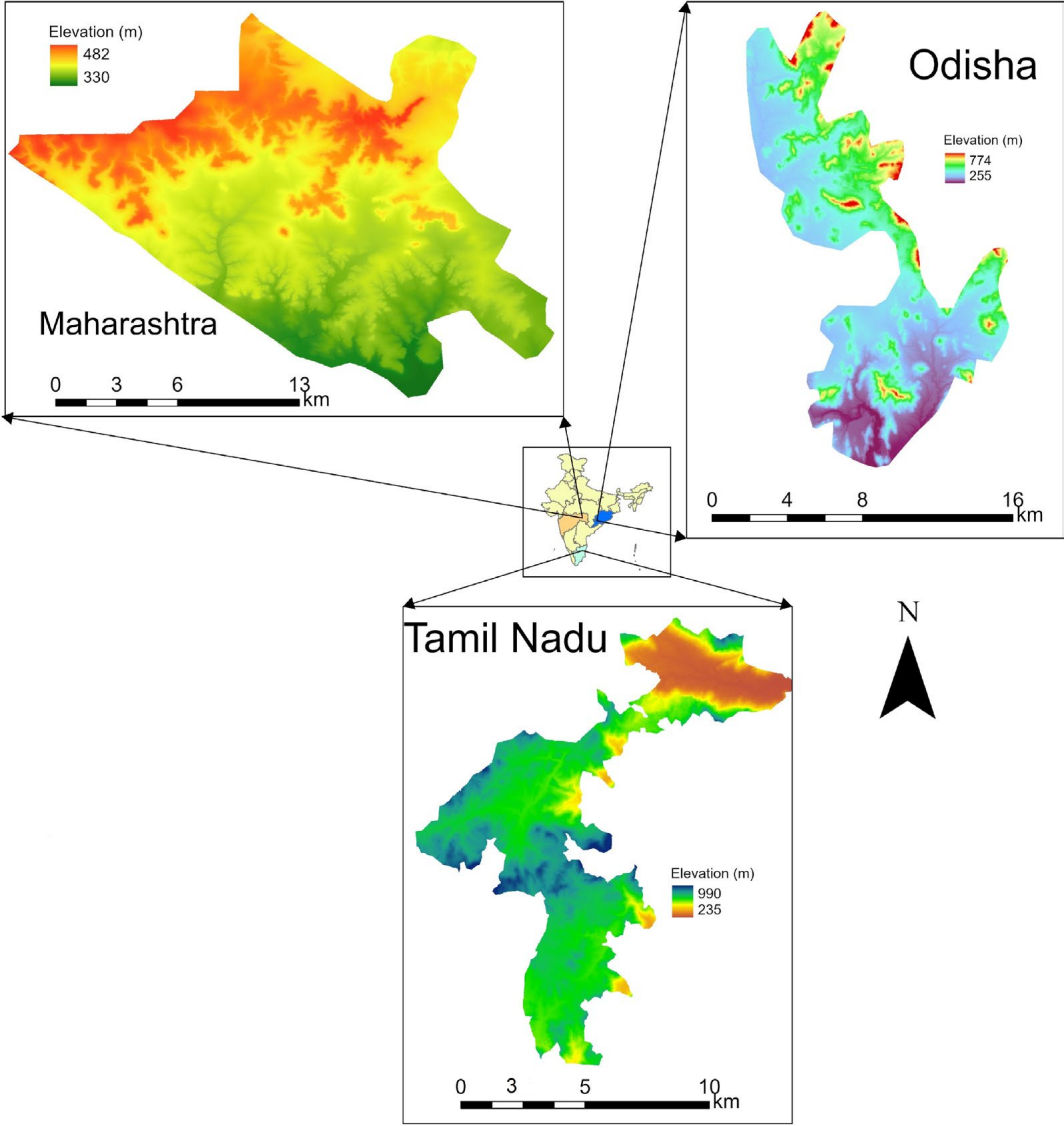
Study area of Maharashtra, Odisha, and Tamil Nadu State, India, shown with LiDAR DEM as the background.

In this study, the TL framework was implemented across three experimental catchments in India (Fig. 1) each exhibiting distinct hydro-geomorphic and climatic characteristics. The following paragraphs describe these catchments in detail.

### Catchment 1 - Odisha ($OD$)

The first experimental catchment, hereafter referred to as E1, is located in the Rayagada district of $OD$, which forms part of the Eastern Ghats highlands. It lies between latitudes 19$^{\circ }$22’ N and 19$^{\circ }$35’ N and longitudes 83$^{\circ }$18’ E and 83$^{\circ }$26’ E. The district is bounded by Gajpati in the east, Koraput and Kalahandi in the west, Andhra Pradesh in the south, and Kalahandi in the north. This region forms part of the Vamsadhara and Nagavali sub-basins, which are the major rivers in this region. The Rayagada district has predominantly rugged and undulating terrain, with elevation ranging between 250 m to 775 m. It is characterized by steep forested hills, dissected plateaus, and narrow valleys. In terms of geology, this region is dominated by the Precambrian Eastern Ghat subgroup of crystalline rocks, overlain in places by Quaternary alluvium. The soils are primarily red sandy loams, lateritic upland soils, and alluvial deposits along river valleys. Due to continuous deforestation and erosion in this region, organic-rich topsoil has been severely depleted.

The Rayagada district receives an average annual rainfall varying between 1031 mm and 1569 mm, predominantly during the southwest monsoon from June to October. Despite the high quantum, the rainfall distribution is highly erratic and uneven. As a result, floods and droughts occur regularly with varying intensity. Moreover, due to this variation in rainfall, streams exhibit high runoff during monsoon months but significantly reduced flow in the dry season, which in turn influences soil erosion and groundwater recharge dynamics. The catchment exhibits diverse LULC, dominated by forests (38%), agricultural land (26%), and scrubland (26%), with other classes such as barren rocks, stony waste, settlements, and wetlands occupying minor areas.

### Catchment 2 - Maharashtra ($MH$)

The second experimental catchment, hereafter referred to as E2, is located in the Amravati district of $MH$, within the Vidarbha region. It lies between latitudes 24$^{\circ }$11’ N and 24$^{\circ }$17’ N and longitudes 85$^{\circ }$18’ E and 85$^{\circ }$32’ E. The district is bounded by Madhya Pradesh to the north, Nagpur and Wardha to the east, and Yavatmal, Akola, and Buldhana to the south and southwest. The terrain consists of broad plains interspersed with the Melghat hilly area of the Satpura range to the north, with elevations reaching up to about 480 m above mean sea level. The district is drained by the Wardha, Purna, and Tapi rivers, along with several seasonal streams that carry flashy discharge during the monsoon, but remain dry for most of the year.

The mean annual rainfall is about 1050 mm, concentrated mainly during the southwest monsoon (June–September). Geologically, the region is dominated by basaltic lava flows of the Deccan Traps, covering nearly three-fourths of the area, while the remaining portions are underlain by alluvium, Gondwana sediments, and unclassified metamorphic rocks. The LULC of the catchment shows moderate variation, with moderately dense forest covering the largest portion (45%), followed by agricultural land (20%). Smaller areas are occupied by settlements, barren land, horticulture, and water bodies. The soils are primarily medium to deep black cotton soils and red loamy soils of clayey texture. Despite receiving considerable rainfall, rapid surface runoff and limited infiltration due to basaltic terrain result in low groundwater recharge potential.

### Catchment 3 - Tamil Nadu ($TN$)

The third experimental catchment, hereafter referred to as E3, is located in the Polur Taluk of Vellore district, Tamil Nadu. It lies between latitudes 12$^{\circ }$38’ N and 12$^{\circ }$51’ N and longitudes 78$^{\circ }$55’ E and 79$^{\circ }$4’ E. Vellore lies in the northern part of Tamil Nadu, sharing its northern boundary with the Chittoor district of Andhra Pradesh. It is bordered by Thiruvallur and Kancheepuram districts to the east, Tiruvannamalai to the south, and Dharmapuri to the west. The terrain consists of a combination of undulating plains and rugged hill ranges forming part of the Eastern Ghats, reaching elevations up to 990 m. The area is drained from west to east by the Palar River and its tributaries–Pennar, Cheyyar, and Pambar–which exhibit flashy discharge during the monsoon and remain mostly dry during the rest of the year. The mean annual rainfall of the region is approximately 885 mm, primarily received during the northeast (October – December) and southwest monsoon (June – September) seasons.

Geologically, the catchment is underlain by Archaean crystalline rocks such as charnockites, granulites, gneisses, and quartzites, intruded by dolerite dykes, with Gondwana shales and sandstones occurring in the eastern part. The LULC of the catchment exhibits considerable variation, with agricultural land (32%) and forested areas (57%) dominating the landscape. Smaller areas are occupied by plantations, scrubland, settlements, barren rock, stony waste, and wetlands ($<$5%), reflecting the heterogeneous land use and vegetation cover across the catchment. The soils range from red and sandy loams on uplands to clayey and black cotton soils in valleys. Despite moderate rainfall, the region faces challenges of high runoff, limited infiltration, and seasonal water scarcity, making it a suitable site for assessing small-scale RWH interventions.

### Datasets

The selection of datasets was guided by the Food and Agriculture Organisation framework for evaluating RWH structures, which emphasizes climate, topography, hydrology, agronomy, and soil–geological characteristics. Accordingly, parameters such as slope, elevation, stream flow, LULC, soil texture, lithology, and geomorphology were considered in this study. For the three study catchments (E1, E2, and E3), the above mentioned data were obtained from various authoritative sources. The details of the datasets and their sources are summarized in Table 1. The stream gauge data were used to calibrate and validate GR4J^27^ rainfall–runoff model for simulating catchment streamflow across all three catchments. Field data were collected by Water and Power Consultancy Services Limited (WAPCOS Ltd.), an Indian consulting firm. A high-resolution (30 cm) LiDAR DEM was utilized by WAPCOS Ltd. to delineate and identify potential micro–soil and water conservation sites within forested regions, supported by reconnaissance surveys and field verification. Across the three catchments, 1345 (E1), 1,063 (E2), and 1,325 (E3) ground-verified RWH structures were considered for the analysis in this study.

**Table 1:** Summary of datasets and data sources used for all three catchments (E1–OD, E2–MH, and E3–TN).

Catchment	Category	Source
E1, E2, E3	Soil	Bhuvan portal^21^
	LULC	Prepared by Geokno India Pvt. Ltd. for the Forest Department using aerial photographs captured during LiDAR point cloud acquisition
	Elevation	10-m resolution LiDAR DEM
	Geology	^22^ (GSI)
	Geomorphology	GSI
	Potential Evapotranspiration (PET)	ERA5 hourly re-analysis data^23^
	Temperature	Indian Meterological Department (IMD) gridded dataset (1$^{\circ } \times 1^{\circ }$ resolution)^24^
E1	Precipitation	Kalyan Singpur (318198317) and Muniguda (318198318) stations, IMD Pune^25^
	Stream gauge	Thotapalli (318188362), data from 2000–2020 obtained from^26^
E2	Precipitation	Amravati, IMD Pune^25^
	Stream gauge	Hivra, data from 1991–2019 obtained from CWC
E3	Precipitation	Arni, IMD Pune^25^
	Stream gauge	Maragal, data from 1980-2018 obtained from CWC

## Methodology

**Fig. 2:**
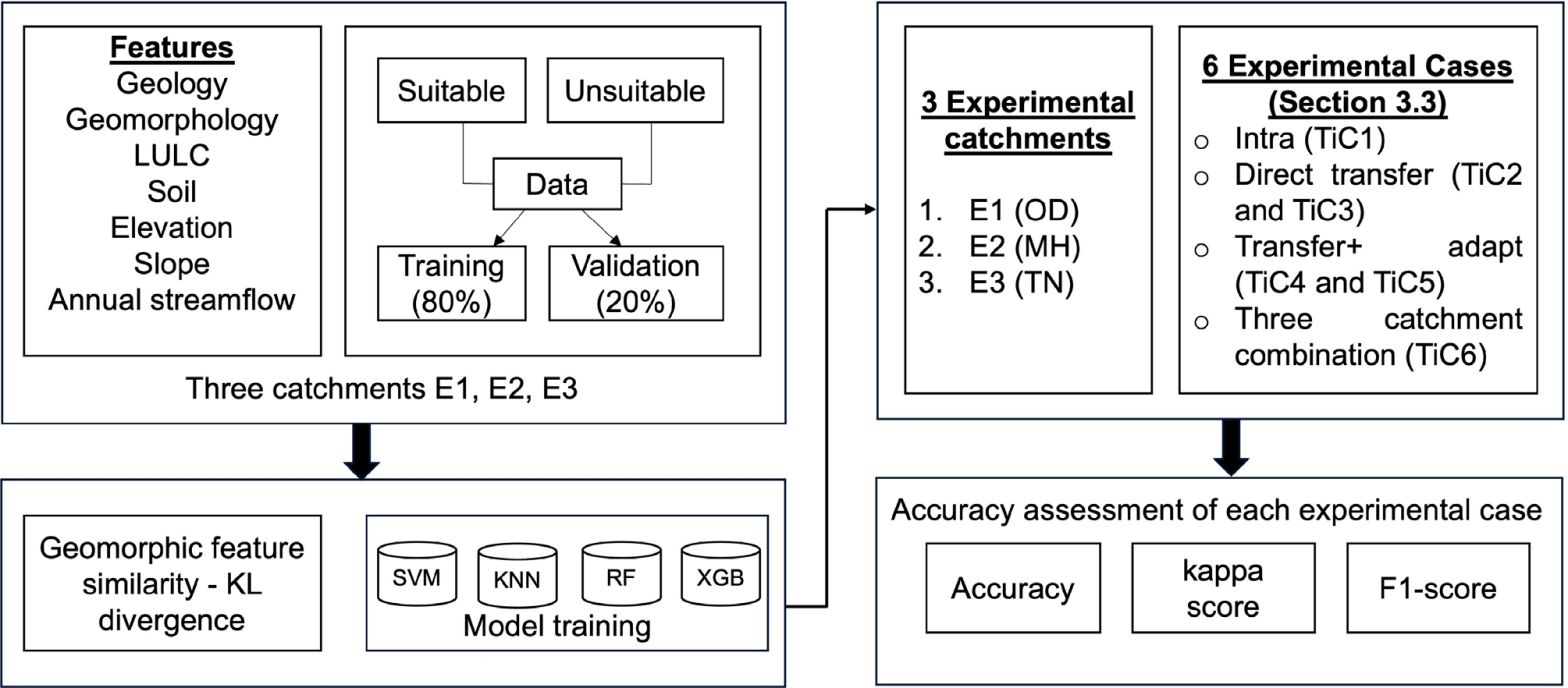
Workflow showing the utilization of different raw data, input thematic data layers, modelling processes, and the resultant outputs for validating the accuracy of each experimental case.

A structured workflow was developed to implement the TL approach for identifying suitable RWH sites as shown in Fig. 2. The workflow integrates four key components: (i) data preparation and feature generation, (ii) hydro-geomorphic similarity assessment, (iii) machine learning model development, and (iv) transfer-learning-based experimental evaluation.

**Fig. 3:**
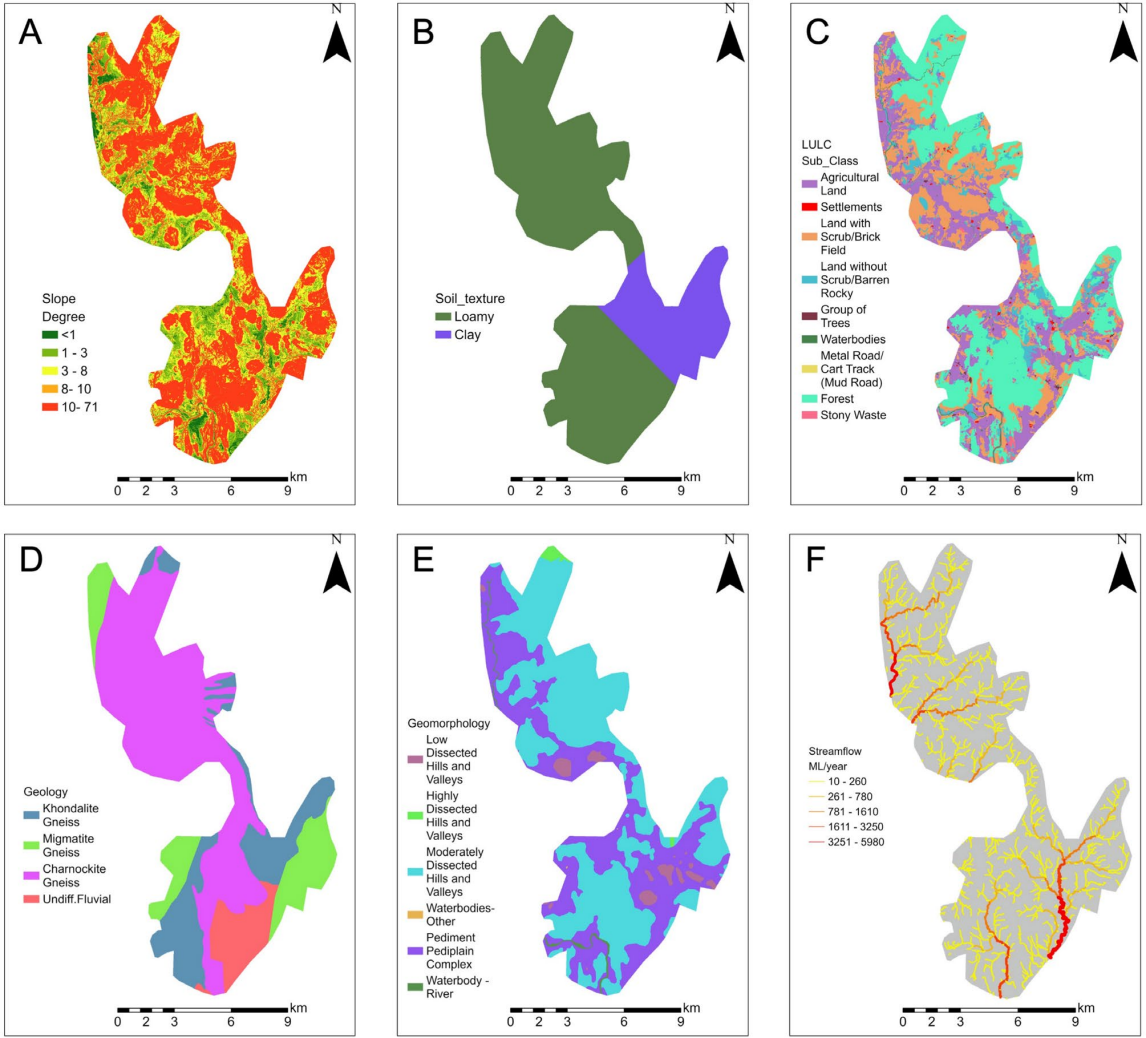
Thematic layers of RWH influencing factors for E1 catchment: (**A**) Slope, (**B**) Soil texture, (**C**) LULC, (**D**) Geology, (**E**) Geomorphology, (**F**) Streamflow. The maps were generated using ArcGIS Pro version 2.9 (https://www.esri.com).

**Fig. 4:**
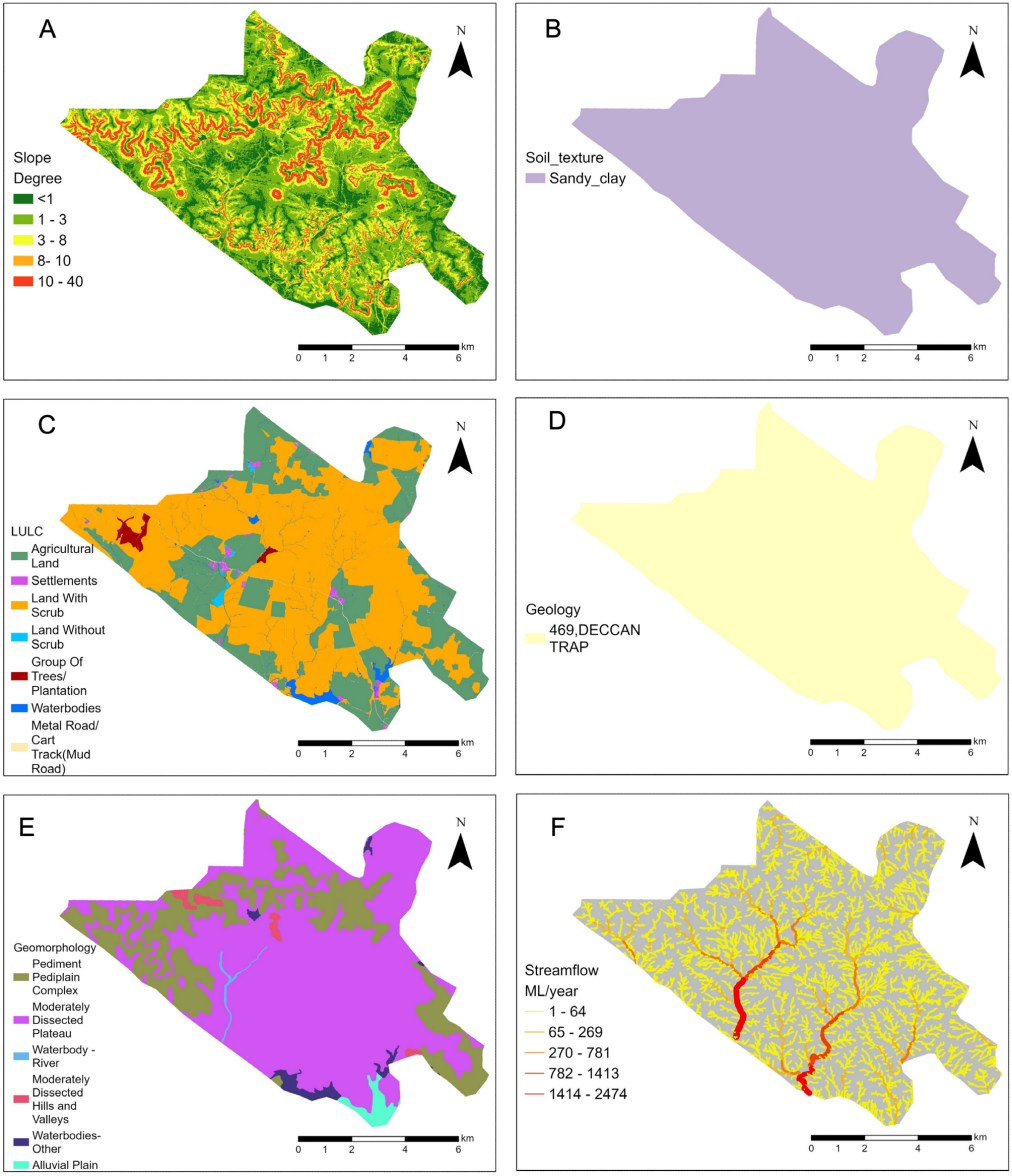
Thematic layers of RWH influencing factors for E2 catchment: (**A**) Slope, (**B**) Soil texture, (**C**) LULC, (**D**) Geology, (**E**) Geomorphology, (**F**) Streamflow. The maps were generated using ArcGIS Pro version 2.9 (https://www.esri.com).

**Fig. 5:**
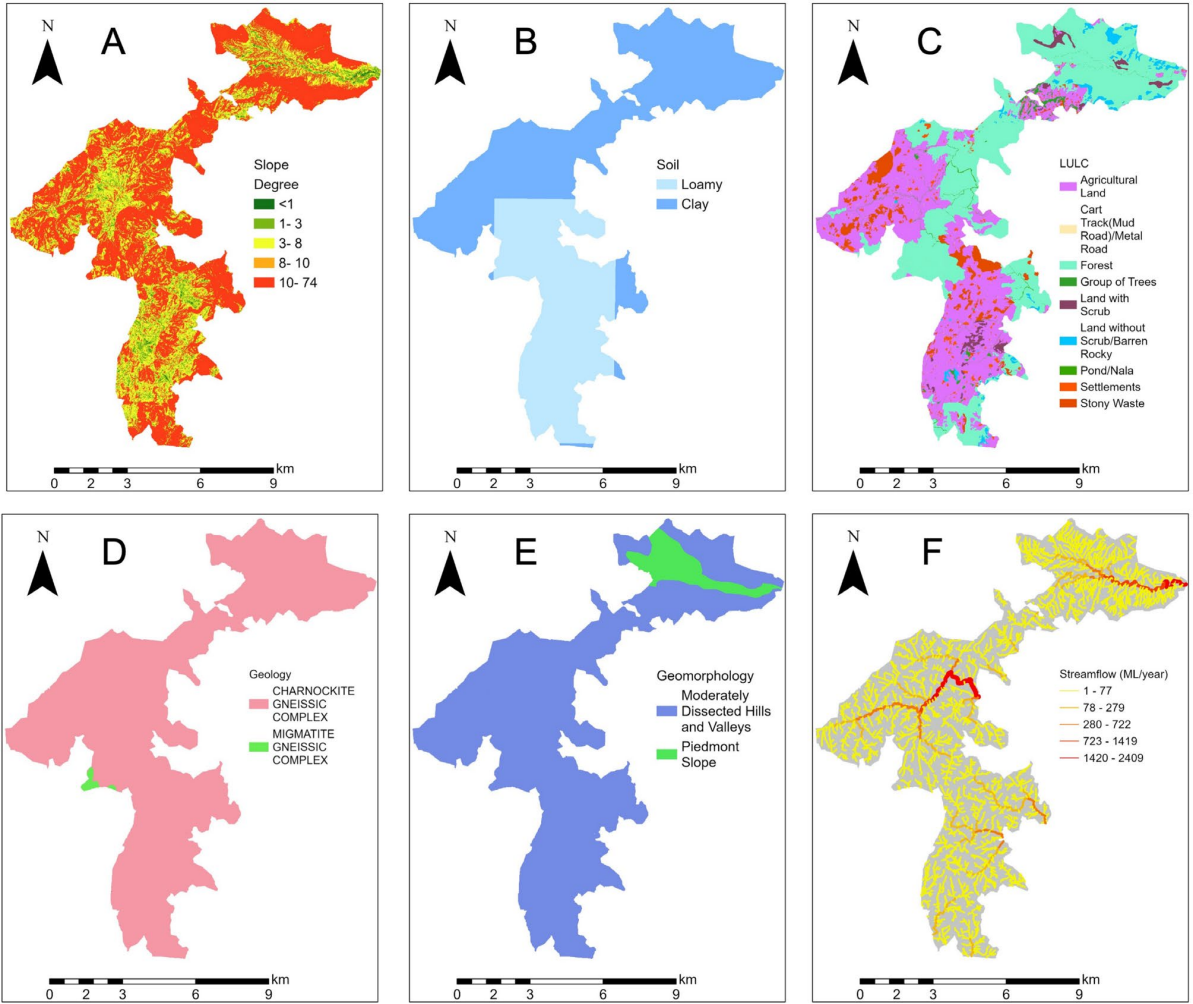
Thematic layers of RWH influencing factors for E3 catchment: (**A**) Slope, (**B**) Soil texture, (**C**) LULC, (**D**) Geology, (**E**) Geomorphology, (**F**) Streamflow. The maps were generated using ArcGIS Pro version 2.9 (https://www.esri.com).

For all three catchments (E1, E2, and E3), raw datasets–including the DEM, geological and soil maps, precipitation, temperature, PET, and ground-verified RWH locations–were compiled. The DEM was pre-processed to remove sinks and ensure hydrological consistency. The spatial data were then processed to generate thematic input layers such as elevation, slope, and hydrological factors required for analysis. All GIS operations, including DEM pre-processing, thematic layer preparation, and the generation of maps (Figs. 3, 4, and 5), were carried out using ArcGIS Pro version 2.9 (https://www.esri.com). Catchment-scale annual streamflow was simulated using the GR4J rainfall–runoff model.

The field-based RWH data were curated through a systematic process that included: (i) extraction and organization of WAPCOS field-verified locations, (ii) validation of the suitable and unsuitable sites using field observations, (iii) applying annual streamflow thresholds, and (iv) incorporating additional unsuitable sites from satellite imagery to improve heterogeneity. The final dataset was labeled and split into training (80%) and validation (20%) subsets.

To quantify inter-catchment similarity, KL divergence was computed for selected geomorphic and hydrological features, providing a measure of distributional differences between source and target catchments and informing transferability. Subsequently, four machine learning models–SVM, KNN, RF, and XGB–were trained under multiple experimental scenarios. A total of six experimental cases were designed for each target catchment, including intra-catchment training, direct transfer, transfer with adaptation, and multi-catchment training. Finally, model performance across all experimental cases was evaluated using standard metrics, including overall accuracy, F1-score, and Cohen’s $\kappa$ coefficient.

### Influential factors

The location of RWH structures is strongly influenced by biophysical factors, including elevation, slope, stream flow, soil texture, LULC, geology, and geomorphology. The descriptions of these factors for each catchment are provided in Section 2, and the corresponding thematic maps for all three catchments are presented in Fig. (3, 4, and 5). To characterize rainfall–runoff relationships, meteorological inputs–such as precipitation, temperature, and PET–were used to estimate annual and sub-annual streamflow across the catchments using the GR4J rainfall–runoff model^27^. The model was calibrated and validated with discharge data from the respective stream gauges through Markov Chain Monte Carlo (MCMC) sampling^28,29^. Simulated annual runoff at each catchment outlet was then integrated with a GIS-based flow accumulation approach^30,31^ to generate spatially distributed annual water volumes, capturing both temporal and spatial variability (Fig. 3F, 4F, 5F). Streamlines with less than $1 - 10\ \mathrm {ML/year}$ are excluded to emphasize locations suitable for loose boulder check dams and gabion dams.

### Datasets

For all three catchments, the initial set of sites identified by WAPCOS through ground surveys was classified as suitable or unsuitable based on field verification and historical records of existing RWH structures. Suitable locations corresponded to stream-based structures such as gabions, anicuts, and loose stone check dams, whereas unsuitable sites included locations for percolation tanks and sunken ponds. To ensure that the curated dataset captured the heterogeneity of terrain, hydrological, and spatial conditions relevant to RWH structures, the curation followed two systematic refinement steps:

1. Hydrological refinement: Annual streamflow (SF) thresholds were applied to WAPCOS sites to retain only locations consistent with the design capacity of 50–100 ML/year (^32^).
2. Terrain, construction feasibility, and spatial refinement (unsuitable class enhancement): To enhance heterogeneity particularly in the unsuitable class–additional unsuitable sites were identified from satellite imagery based on steep terrain, soil constraints, or construction infeasibility. Finally, random off-stream points were incorporated to broaden spatial coverage and reduce sampling bias.

Across the three catchments, the curated datasets consists of:

(i) E1 – OD: Initially 751 WAPCOS sites (387 suitable, 364 unsuitable); after SF-based filtering, 102 suitable and 364 unsuitable sites were retained, supplemented with 97 satellite-derived unsuitable locations and 497 random off-stream points; (ii) E2 – MH: Initially 503 WAPCOS sites (291 suitable, 210 unsuitable); after filtering, 82 suitable and 210 unsuitable sites were retained, augmented by 68 satellite-derived unsuitable sites and 494 random off-stream points; and (iii) E3 – TN: Initially 602 WAPCOS sites (261 suitable, 341 unsuitable); after filtering, 60 suitable and 341 unsuitable sites were retained, supplemented with 87 satellite-derived unsuitable sites and 636 random off-stream points (Table 2). Increasing the number of additional unsuitable locations beyond these levels did not result in any substantial improvement in model performance; hence, the above configuration was considered optimal for all subsequent analyses. This structured curation process ensured the creation of a heterogeneous and representative dataset for robust ML modelling.

**Table 2:** Summary of RWH site curation for all three catchments.

Site Category	E1 ($OD$)	E2 ($MH$)	E3 ($TN$)
Initial WAPCOS Sites	751	503	602
Suitable Sites after SF-filtering	102	82	60
Unsuitable Sites after SF-filtering	364	210	341
Additional Unsuitable Sites (satellite-derived)	97	68	87
Additional Unsuitable Sites (random off-stream)	497	497	636
Total (Suitable + Unsuitable)	1060	854	1124

### Experimental cases and naming convention

In this study, three experimental catchments–E1, E2, and E3–were used to design a series of TL experiments aimed at evaluating the feasibility and robustness of cross-catchment knowledge transfer for RWH suitability modelling. Multiple combinations of these catchments were created to systematically examine how well models trained in one catchment generalize to another with different terrain, hydrological, and land-surface characteristics. A structured naming convention was adopted, where “Ti” denotes the target catchment (T1 = E1 ($OD$), T2 = E2 ($MH$), T3 = E3 ($TN$)) and “Cj” denotes the specific transfer or adaptation case (C1–C6). For example, T1C1 represents a case where E1 is the target catchment and C1 corresponds to the first experimental case. The source–target configurations and naming scheme for all experimental cases are summarized in Table 3.

For the first set of experiments, E1 was designated as the target catchment (T1), while the remaining two catchments served as sources. A baseline case (T1C1) was implemented, trained, and validated solely on 20% of E1 data. Direct transfer cases (T1C2 and T1C3) were then performed, where models trained on E2 and E3 were directly validated on 20% of E1 to assess their generalization capability across catchments. To improve the model adaptability, two adaptation cases (T1C4 and T1C5) were implemented, where a small portion of the target catchment data (20% of E1) was added to the source-trained models, resulting in E2 + 20% E1 and E3 + 20% E1, both validated on 20% of E1. This allows recalibration of feature weights to better align with the target characteristics. Finally, a combined case (T1C6) was implemented in which the model was trained using data from all three catchments (E1 + E2 + E3) and validated on 20% of E1 to evaluate the performance when leveraging the full dataset. Equivalent experimental setups were performed for E2 and E3 as target catchments, resulting in a total of 18 experimental cases (6 per target), providing a comprehensive assessment of transfer learning under varying source–target combinations.

**Table 3:** Overview of experimental cases for three catchments (E1, E2, and E3).

Experiment	Target catchment	Source catchment	Case type
T1C1	E1	E1	Intra
T1C2	E1	E2	Direct transfer 1
T1C3	E1	E3	Direct transfer 2
T1C4	E1	E2 + 20% of E1	Transfer + adaptation 1
T1C5	E1	E3 + 20% of E1	Transfer + adaptation 2
T1C6	E1	E1+E2+E3	Three catchment combination
T2C1	E2	E2	Intra
T2C2	E2	E1	Direct transfer 1
T2C3	E2	E3	Direct transfer 2
T2C4	E2	E1 + 20% of E2	Transfer + adaptation 1
T2C5	E2	E3 + 20% of E2	Transfer + adaptation 2
T2C6	E2	E2+E3+E1	Three catchment combination
T3C1	E3	E3	Intra
T3C2	E3	E1	Direct transfer 1
T3C3	E3	E2	Direct transfer 2
T3C4	E3	E1 + 20% of E3	Transfer + adaptation 1
T3C5	E3	E2 + 20% of E3	Transfer + adaptation 2
T3C6	E3	E3+E1+E2	Three catchment combination

### Kullback-Leibler divergence

To quantify the similarity between source and target catchments, the KL divergence was computed for each influencing feature. It quantifies the difference between two probability distributions and, in the context of TL for RWH, assesses the degree of dissimilarity between the feature distributions of the source and target catchments. A higher divergence value indicates greater variation in the underlying feature characteristics, implying reduced similarity between the catchments.

The KL divergence between two discrete probability distributions $P$ and $Q$ is defined as:

1 $$\begin{aligned} D_{KL}(P \parallel Q) = \sum _{i} P(i) \log \frac{P(i)}{Q(i)} \end{aligned}$$

where $P$ and $Q$ represent the probability distributions of the source and target catchment, respectively, and $i$ indexes the feature outcomes. KL divergence provides a directional measure of dissimilarity between two feature distributions; therefore,

$$D_{KL}(P \parallel Q) \ne D_{KL}(Q \parallel P)$$

This asymmetry is informative, as it reflects how well a source catchment can approximate the target distribution, which is directly relevant for transfer learning feasibility. Lower $D_{KL}$ values indicate higher feature similarity between the source and target catchments, while higher values signify greater divergence.

In this study, KL divergence was evaluated for all possible source–target combinations among the three catchments, resulting in six pairwise comparisons: E1–E2, E2–E1, E1–E3, E3–E1, E2–E3, and E3–E2. This analysis provides a quantitative basis for identifying which catchments exhibit similar hydrological and biophysical conditions.

### ML models and techniques

Four ML models were employed in this study, each selected for its distinct capability to handle complex geospatial and environmental datasets, as illustrated in Fig. 2. Ground-verified RWH locations, as discussed in the methodology section, were categorized into two reference classes (suitable and unsuitable), while biophysical influencing factors derived from thematic layers served as predictor variables. Each model was trained separately for the 18 experimental cases and evaluated on an independent test set to generate corresponding outputs.

The models were chosen for their complementary learning mechanisms and proven applicability in hydrology and environmental mapping. The SVM constructs an optimal hyperplane to separate classes, making it particularly effective in high-dimensional feature spaces^4^. The RF algorithm, an ensemble of decision trees, enhances model stability and reduces overfitting by aggregating predictions from multiple randomized trees^10^. The XGB model employs sequential boosting to iteratively refine weak learners, improving predictive accuracy–especially for imbalanced or noisy datasets^33^. The KNN algorithm classifies each data point based on the majority class of its nearest neighbors, effectively capturing localized spatial patterns in the data^15^.

To ensure robust and unbiased model performance, the Synthetic Minority Over-sampling Technique (SMOTE) was applied to address class imbalance in the training sets, preventing the models from being biased toward the majority (unsuitable) class, since the number of suitable sites was considerably lower in all three catchments. All input features were standardized using a standard scaler to ensure equal contribution of variables during model training. Hyperparameter tuning was conducted using GridSearchCV^34^, enabling systematic exploration of parameter combinations to identify the optimal configuration for each model. Each model was trained and validated using 100 randomized stratified train–test splits, ensuring that the original ratio of suitable to unsuitable sites was maintained in every split. This strategy enables performance assessment across multiple data partitions, yielding more reliable estimates of accuracy and variability while minimizing the impact of any single, potentially unrepresentative split.

### Model performance

Model performance was evaluated using a confusion matrix (Table 4) that defines true positives ($TP$), false positives ($FP$), false negatives ($FN$), and true negatives ($TN$). In this study, $TP$ and $FP$ correspond to correctly and incorrectly predicted suitable sites, respectively, whereas $TN$ and $FN$ denote correctly and incorrectly predicted unsuitable sites, respectively. These metrics provide a quantitative assessment of the classification model’s predictive performance, accounting for both correct and incorrect predictions across suitable and unsuitable sites.

**Table 4:** Confusion matrix explaining the true positives ($TP$), true negatives ($TN$), false positives ($FP$), and false negatives ($FN$).

		Classified by the algorithm	
		Suitable	Unsuitable
On-ground	Suitable	$TP$	$FP$
	Unsuitable	$FN$	$TN$

Quantitative evaluation was carried out using overall accuracy ($OA$), F1-score, and Cohen’s $\kappa$ coefficient^35^, defined as:

2 $$\begin{aligned} & OA = \frac{TP + TN}{TP + FP + TN + FN} \end{aligned}$$

3 $$\begin{aligned} & Precision = \frac{TP}{TP+FP}\ \end{aligned}$$

4 $$\begin{aligned} & Recall = \frac{TP}{TP+FN}\ \end{aligned}$$

5 $$\begin{aligned} & F1\text {-score} = 2 \times \frac{Precision \times Recall}{Precision + Recall} \end{aligned}$$

6 $$\begin{aligned} & \text {Cohen's } \kappa = \frac{(p_o - p_e)}{(1 - p_e)} \end{aligned}$$

where $p_o$ and $p_e$ represent the observed and expected probability of agreement, respectively.

## Results

This section presents the results of KL divergence and the performance outcomes of all experimental cases designed for the TL approach for RWH suitability mapping using SVM, RF, XGB, and KNN classifiers.

### KL divergence

The computed KL divergence values for all influencing feature layers across the six source–target catchment pairs are summarized in Table 5.

**Table 5:** KL divergence values for seven influencing factors across all source–target catchment combinations.

Factors	KL divergence					
	E1-E2	E2-E1	E1-E3	E3-E1	E2-E3	E3-E2
Geology	5.52	6.90	3.92	1.49	6.64	6.97
Geomorphology	0.15	0.15	2.63	1.30	0.97	1.17
LULC	1.14	0.54	2.81	1.19	1.91	1.74
Soil	6.22	6.91	0.41	0.46	6.07	6.97
Slope	0.53	0.52	0.03	0.03	0.73	0.90
Elevation	0.56	0.22	0.83	1.00	3.64	1.64
Streamflow	0.01	0.01	0.01	0.01	0.02	0.02

Geology and soil exhibit the highest divergence values, exceeding 5.0 in several combinations (E1-E2, E2-E1, E2-E3, and E3-E2), indicating pronounced differences in subsurface characteristics among catchments. Geomorphology and LULC display moderate divergence, typically ranging between 0.5 and 2.8, with higher values observed for the E1–E3 pair, suggesting moderate variation in surface form and land-use patterns. In contrast, E3–E1 shows generally low divergence across all parameters (mostly 1.5), indicating closer similarity when transferring from E3 to E1. This highlights the inherent asymmetry of KL divergence ($D_{KL}(P \parallel Q) \ne D_{KL}(Q \parallel P)$), where the direction of transfer affects the perceived similarity. Slope and elevation show low divergence (mostly 1.0), except for elevation in the E2–E3 pair (3.64). Streamflow exhibits the lowest divergence across all combinations, indicating comparable hydrological response patterns. Overall, E2–E3 and E3–E2 register the highest aggregate divergence, E1–E3 shows moderate variability, and E1–E2, E2–E1, and E3–E1 demonstrate relatively higher similarity among the three experimental catchments. These patterns suggest that cross-catchment transfer learning is likely more reliable for pairs with lower divergence, while higher divergence (especially in geology and soil) could hinder model generalization.

### ML performance catchment E1 ($OD$)

**Fig. 6:**
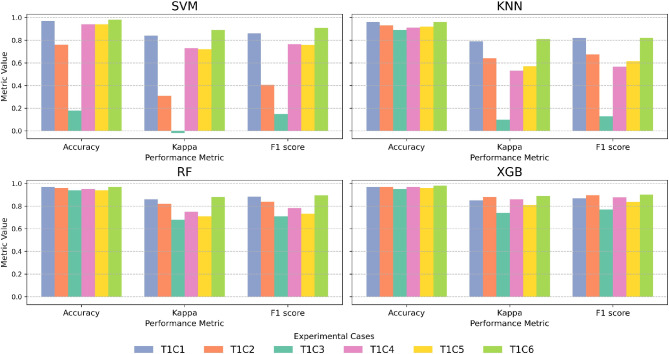
Performance of four ML models (SVM, KNN, RF, XG Boost) across the six cases for E1 catchment, showing variations in accuracy, kappa, and AUC metrics.

For the E1 catchment, model performance across the six experimental cases (Fig. 6) revealed distinct differences in transfer effectiveness. In case T1C3, the direct transfer from E3 to E1 resulted in the weakest performance across all models, with near-zero kappa values – SVM (0.02), KNN (0.10), RF (0.68), and XGB (0.74), and low F1-scores ranging from 0.13 (KNN) to 0.77 (XGB). Similarly, in T1C2, the direct transfer from E2 to E1 showed moderate performance, achieving kappa values of 0.31–0.88 and F1-scores between 0.41 (SVM) and 0.90 (XGB).

In contrast, the target-trained baseline case (T1C1) achieved the highest performance across all classifiers, with accuracy between 0.96 and 0.97, kappa values from 0.79 to 0.86, and F1-scores of 0.82–0.88. When limited E1 samples were integrated with the source data from E2 and E3 (T1C4 and T1C5), the models showed a clear adaptability gain, improving the kappa values to 0.53–0.86 and F1-scores to 0.56–0.88. The multi-source transfer case (T1C6) produced the most stable and balanced results, maintaining high accuracy (0.96–0.98), kappa (0.81–0.89), and F1-score (0.82–0.90) for SVM, RF, XGB, and KNN models.

**Fig. 7:**
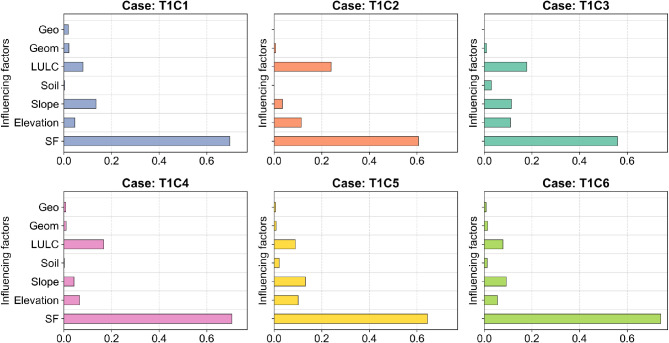
Feature importance of RF model across cases for OD catchment (E1).

The feature importance analysis of all the models for the E1 catchment (Fig. 7) revealed that streamflow was the most dominant predictor, contributing over 60–70% of the model importance across all experimental cases. Next, the elevation consistently ranked as the second most influential feature (10–20%), followed by slope. The remaining variables–geology, soil, and LULC–showed comparatively minor contributions ($<$10%) within the E1 catchment. The feature importance distributions of the other models (SVM, KNN, and XGB) are provided in the Supplementary Material (Figure S1, S2, and S3).

For the transfer scenarios, the feature importance of the RF model exhibited distinct variations. In the direct transfer cases (T1C2 and T1C3), where models trained on E2 and E3 were applied to the E1 catchment, the feature importance values displayed greater dispersion, with inconsistent weighting among geomorphology, soil, and LULC. This variability indicates a limited alignment between the feature–response relationships learned from the source basins and the unique hydro-geomorphic characteristics of E1. In contrast, the adapted cases (T1C4 and T1C5) showed partially stabilized feature importance patterns among streamflow, LULC, and elevation, while the contributions of other features became more uniform. This stabilization reflects the model’s improved internal representation of local hydrological–terrain interactions following adaptation. Notably, in the combined case (T1C6), the model achieved the most balanced distribution of feature importance and the highest predictive performance (kappa> 0.75, F1-score > 0.9), confirming that the samples from multiple sources enhances both transferability and robustness.

### ML performance catchment E2 ($MH$)

**Fig. 8:**
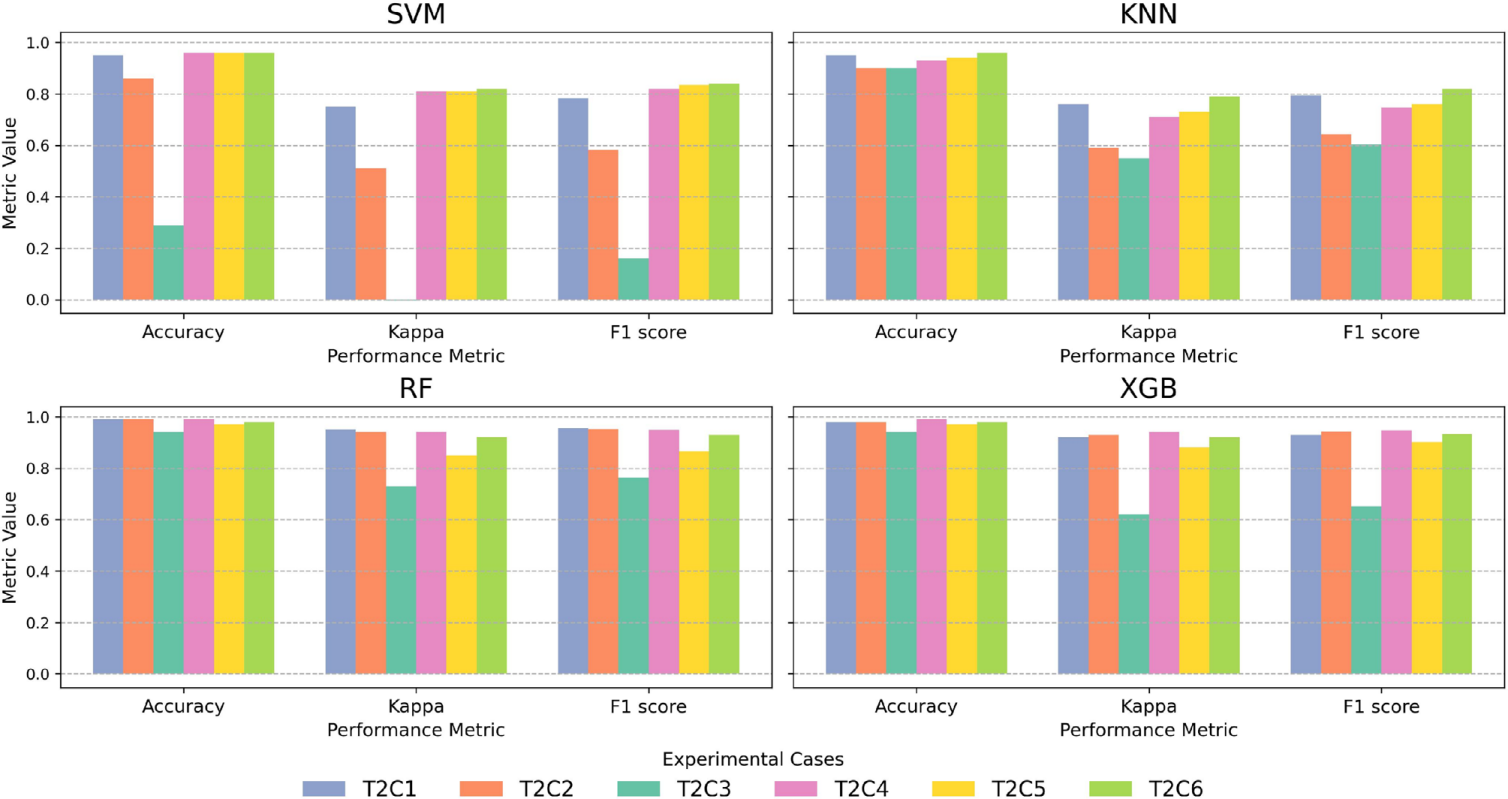
Performance of four ML models (SVM, KNN, RF, XG Boost) across cases for MH catchment (E2), showing variations in Accuracy, Kappa, and AUC, with combined datasets giving the most robust results.

For the E2 catchment, model performance across the six experimental cases (Fig. 8) revealed consistently strong outcomes across most cases. The target-trained case (T2C1) achieved the highest performance, with the RF model yielding an accuracy of about 0.99, a kappa score of approximately 0.95, and an F1-score of 0.96. The SVM and XGB models also performed comparably well, both maintaining accuracy above 0.95 and F1-scores above 0.90, while KNN showed slightly lower yet reliable results (F1-score = 0.79).

Direct transfer from E1 to E2 (T2C2) and from E3 to E2 (T2C3) caused a moderate decline in performance, with kappa values decreasing to between 0.50 and 0.75 across models and F1-scores dropping to around 0.16–0.65. Among all models, SVM and RF maintained better generalization in these transferred cases, whereas KNN exhibited more variability, with kappa ranging from 0.55 to 0.80. When limited E2 data were integrated with data from source catchments E1 and E3 (T2C4 and T2C5), the models demonstrated clear adaptability, with F1-scores recovering to 0.75–0.90 and kappa values exceeding 0.80. The multi-source transfer configuration (T2C6) provided the most stable and balanced performance across all models, maintaining accuracy above 0.95, kappa above 0.90, and F1-scores close to 0.93, confirming that leveraging multiple data sources enhances transfer robustness and prediction reliability for the E2 catchment.

**Fig. 9:**
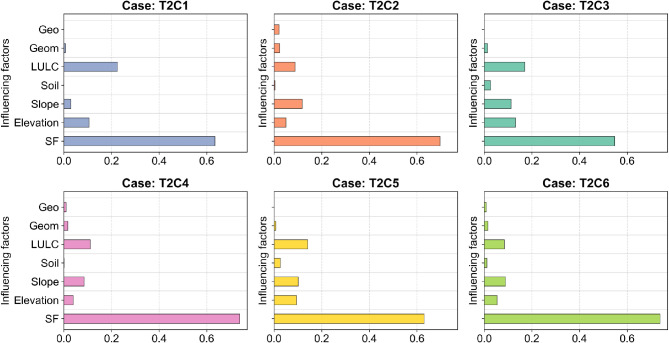
Feature importance of RF model across cases for MH catchment (E2).

The feature importance analysis for E2 catchment (Fig. 9) shows streamflow again as dominating the model predictions, contributing 60–70% of the total importance. Unlike E1, LULC ranked as the second most influential factor (15–20%), while elevation, slope contributed modestly (each $<$ 10%), and the other variables – geology, soil, and geomorphology – showed minimal contributions. The feature importance distributions of the other models (SVM, KNN, and XGB) are provided in the Supplementary Material (Figure S4, S5, and S6).

In the RF model feature importance, notable variations were observed across the transfer scenarios. In direct transfer scenarios (T2C2 and T2C3), the model’s feature weighting became less stable, with slight increases in the importance of LULC and elevation–likely reflecting the model’s attempt to compensate for domain mismatch. However, in the adapted cases (T2C4 and T2C5), the dominance of streamflow and LULC was re-established, with stable contributions from topographic factors. In particular, the combined case (T2C6) achieved a balanced representation of hydrological, terrain, and land-cover features, corresponding with higher predictive accuracy and generalization performance. This result also aligns with the overall performance observed in the E1 catchment.

### ML performance catchment E3 ($TN$)

**Fig. 10:**
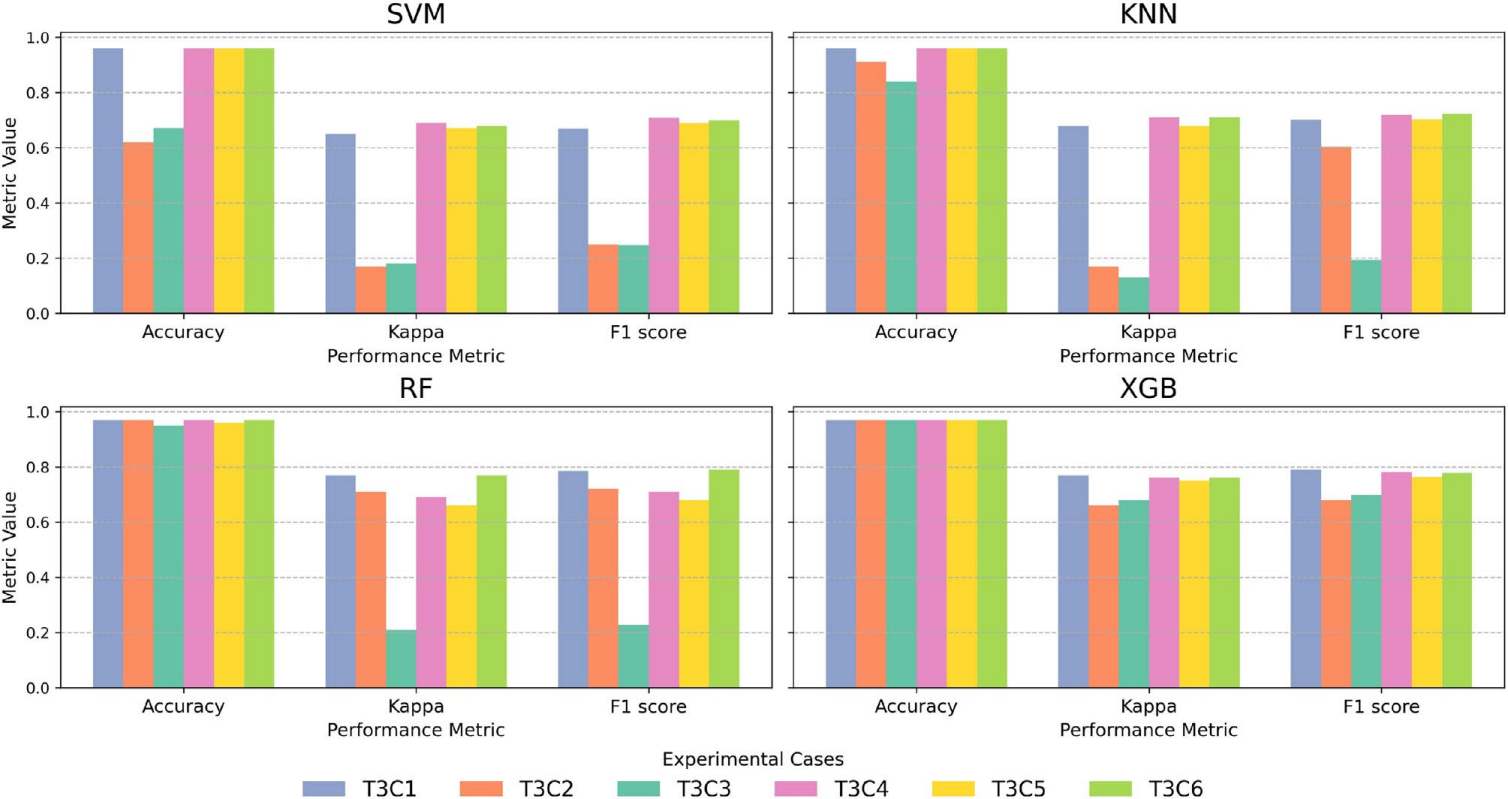
Performance of four ML models (SVM, KNN, RF, XG Boost) across cases for TN catchment (E3), showing variations in Accuracy, Kappa, and F1-score, with combined datasets giving the most robust results.

The model performance across the six experimental configurations for E3 catchment (Fig. 10) revealed pronounced contrasts in transfer effectiveness. The target-trained setup (E3C1) delivered the strongest outcomes, with RF and XGB achieving accuracy between $0.97$–$0.99$, a kappa value of $0.85$–$0.90$, and F1-scores ranging from $0.78$–$0.79$, confirming excellent discriminative capability when trained on native samples.

Conversely, direct transfer from E2 to E3, case T3C2, and E1 to E3, case T3C3, resulted in substantial performance degradation, particularly for SVM and KNN, where kappa declined below 0.20 and F1-scores dropped below 0.25. This sharp reduction highlights the sensitivity of the models to cross-catchment variability in hydrological, geomorphic, and climatic characteristics. When a limited subset of E3 samples was incorporated into the source domain cases – T3C4 and T3C5 – the performance improved significantly, with kappa values increasing to 0.65–0.75 and F1-scores rising to around 0.70, demonstrating the strong positive influence of even minimal target-domain data in guiding feature adaptation. The multi-source hybrid configuration (T3C6) produced the most stable and balanced outcomes for all algorithms, maintaining Accuracy$>$0.95, Kappa$>$0.85, and F1-score$>$0.72. These results collectively affirm that integrating knowledge from multiple basins enhances model generalization and mitigates domain shift, offering a more transferable framework for RWH site prediction under heterogeneous regional conditions.

**Fig. 11:**
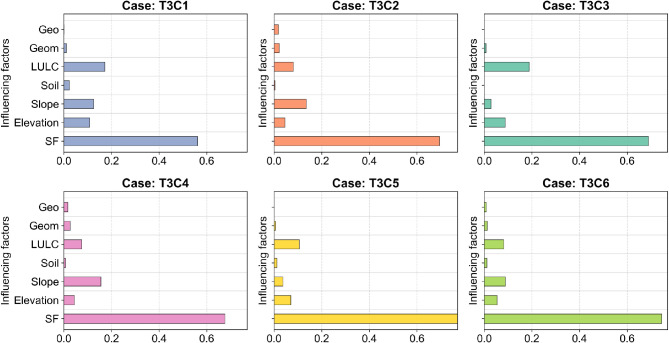
Feature importance of RF model across cases for TN catchment (E3).

In the E3 catchment (Fig. 11), the feature importance distribution of the RF model showed a more balanced pattern. Streamflow remained dominant (approximately 60–65%), but the combined contribution of LULC and topographic features (elevation and slope) accounted for nearly 20–25% of total importance. The remaining features–geology, soil, and geomorphology–showed low but consistent contributions ($<$10%). The relatively higher topographic importance, compared to the E2 catchment, can be attributed to stronger elevation gradients and more defined drainage networks. The feature importance distributions of the other models (SVM, KNN, and XGB) are provided in the Supplementary Material (Figure S7, S8, and S9).

When models are directly transferred from catchment E1, related to case T3C2, the feature importance became stable between streamflow, LULC, and topographic indicators, whereas for the transfer from the E2 catchment related to case T3C3, the feature importance structure was altered, where the LULC became dominant after streamflow dominance. This mismatch suggests that the feature hierarchy learned from external basins fails to align with TN’s local hydrological controls, leading to the observed drop in transfer performance.

However, with limited target-domain adaptation (T3C4 and T3C5), a partial balance in the feature importance ranking was observed. This convergence highlights the models’ capacity to relearn relevant predictors with minimal local target data. In the multi-source configuration (T3C6), the feature-importance patterns became more stable and closely aligned with the target-trained baseline, indicating improved generalization and better feature alignment. Although this behaviour is broadly consistent with the trends observed in the E1 and E2 catchments, the performance patterns reveal an important distinction: while multi-source learning produced consistently strong results in E1 and E2 (F1 = 0.88–0.93), T3C6 achieved comparatively lower scores (F1 = 0.76–0.79). This indicates that despite using the same multi-source training data and achieving improved feature alignment, the generalizability of the multi-source model is still constrained when the target catchment (E3) is hydro-geomorphically more distinct from all other source catchments.

## Discussion

### Geomorphic feature similarity and transfer effectiveness

The geomorphic feature similarity (GFS), quantified using the KL divergence, revealed distinct inter-catchment variations in the similarity of influencing factors, offering a quantitative explanation for the observed differences in TL performance. Higher divergence values denote greater dissimilarity in the underlying feature distributions, particularly for geology and soil, which emerged as the two most divergent factors across basins. For instance, the E3-E2 and E2-E3 pairs exhibited the highest average KL divergence (above 3.0), reflecting the pronounced lithological and geomorphic contrasts between the Deccan basaltic terrain of $MH$ and the crystalline metamorphic formations of $TN$ (e.g., khondalite and charnockite). These contrasts drive differences in permeability, soil formation, and runoff generation processes, ultimately limiting the model’s ability to generalize across such distinct physical environments. Conversely, the E1–E3 and E3–E1 pairs demonstrated relatively lower divergence in geology and soil, as both catchments are predominantly underlain by metamorphic rock assemblages.

Relating the KL divergence to the experimental cases, the high divergence in geology and soil explains the reduced transferability observed in direct transfer (all C2 and C3 cases). For example, in the case T2C3, the model trained on E3 ($TN$) data struggled to generalize to E2 ($MH$) due to substantial differences in lithology and soil permeability–key controls on infiltration and runoff. After adaptation (T2C5), fine-tuning with limited E2 target data enabled the model to realign its feature importance with that of E3, indicating that the adaptation phase effectively reweighted the influence of geology and soil, thereby restoring consistency with the source domain and improving prediction stability. This was reflected in improved F1-score and kappa scores, confirming that feature alignment enhances TL performance.

These divergence and adaptation trends are further corroborated by the RF feature importance patterns. In low-divergence pairs such as E1-E2, the dominant features–streamflow and elevation–jointly accounted for over 70% of model importance, indicating stable hydrological controls and strong feature alignment between domains. In contrast, in highly divergent combinations such as E2-E3 and E3-E2, the feature importance structure became notably unstable across classifiers: geology and LULC gained contribution along with hydrological variables. This redistribution reflects the model’s adaptive response to mismatched environmental conditions, where feature hierarchies are reorganized to accommodate distinct regimes in the target basin.

Collectively, these findings indicate that the degree of divergence in key environmental controls–geology and soil in this study largely determines direct transfer success. When divergence is low to moderate, direct transfer preserves feature–response relationships and can yield acceptable generalization (e.g., E1-E2). Conversely, high divergence in the influencing factors undermines direct transferability (e.g., E3-E2), producing unstable predictions. Crucially, however, adaptation (fine-tuning with a limited amount of target data or using multi-source training) can reweight feature importance and substantially restore predictive stability, even where direct transfer initially fails.

### Relation between GFS, ML model performance, and feature importance

**Fig. 12:**
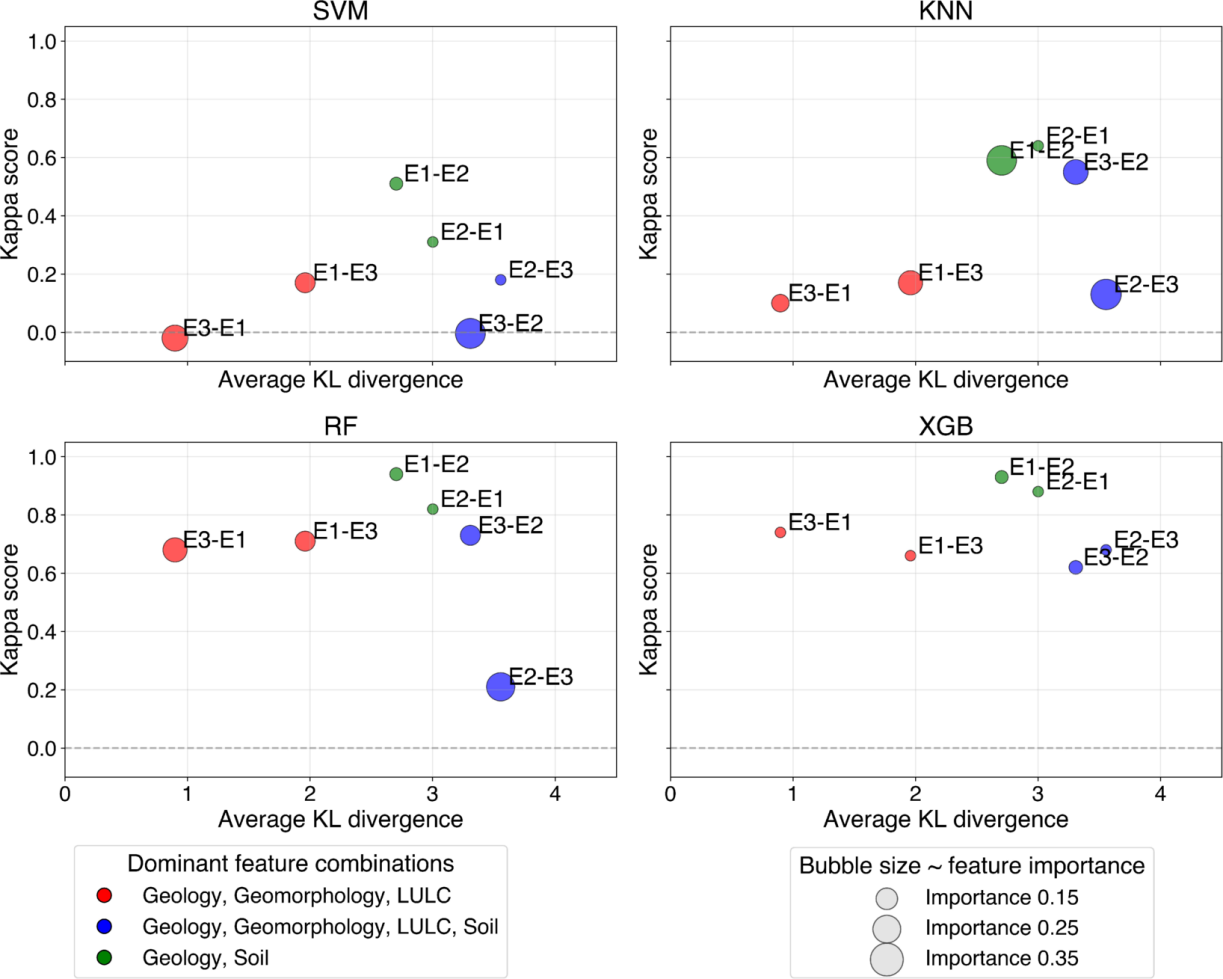
Scatter plot between Kappa Cohen’s value and Feature Divergence shows the relationship between the model’s performance (Kappa score) and the dissimilarity of geospatial features between regions (Average KL Divergence).

To evaluate the TL performance in direct transfer scenarios (Case 2 and Case 3) across the three catchments, the relationship between the ML models, generalized feature similarity (GFS), and feature importance was examined using scatter plots, as shown in Fig. 12. The figure illustrates how the average KL divergence of dominant features relates to transferability performance (kappa score) for each classifier across the six source–target catchment pairs. Each bubble represents a specific transfer direction (e.g., E1-E2), where the bubble color indicates the dominant feature combinations influencing transferability, and the bubble size reflects their relative importance in the respective ML model. Across direct transfer cases, streamflow consistently emerged as the most influential feature. Therefore, to better distinguish the role of other factors, the bubble plot highlights the next most dominant features, since the contribution of streamflow remains comparable across all cases and classifiers.

A consistent inverse relationship between average KL divergence and kappa score is observed across all models – lower divergence values correspond to higher transfer performance. This confirms that when the dominant feature distributions between source and target catchments are more similar, the trained models generalize better during transfer. Conversely, transfers with higher divergence values exhibit a marked decline in kappa score, implying weaker feature–response consistency across domains.

Based on the model-wise interpretation, RF and XGB exhibit the strongest transfer performance, maintaining kappa scores above 0.6 for several pairs despite moderate divergence levels (KL - 2–3). Notably, the E3-E1 pair (red bubble) achieved a kappa of around 0.7–0.8 at low divergence (1.0), while E1-E2 and E2-E1 (green bubbles) also show high performance, reflecting robust feature similarity and model adaptability. Whereas in the case of SVM and KNN models, a sharper decline in the kappa score is observed with increasing divergence, showing limited tolerance to feature distribution shifts. Transfers such as E3-E2 and E2-E3 (blue bubbles) lie at the far right of the plots with low Kappa values ($<$0.3), indicating poor generalization when the feature divergence is high.

For SVM and KNN, the relationship between divergence and transferability is more sensitive and non-linear. Both models exhibit a distinct inverse trend where kappa scores steadily decrease as average KL divergence increases, highlighting their dependence on feature-space consistency between source and target domains. In these models, performance strongly reflects how transferable the underlying feature distributions are. For instance, transfers such as E1-E2 and E2-E1 (green bubbles) achieve moderate Kappa values (>0.5) at intermediate divergence levels (KL - 2.5–3.0), suggesting that when geology and soil remain relatively similar across catchments, even simpler classifiers can retain predictive structure. Conversely, transfers involving E3 (e.g., E3-E2, E2-E3) display large blue bubbles positioned at high divergence with very low Kappa scores ($<$0.3), indicating that pronounced shifts in geomorphology and LULC distributions disrupt the feature–response relationship, limiting adaptation. It is also noteworthy that, due to the directional nature of KL divergence and converse feature similarity, the transfer performance for a pair of catchments (Ei–Ej) is not necessarily the same as that for the reverse direction (Ej–Ei).

The bubble size further clarifies this behavior, smaller or moderately sized bubbles–implying balanced feature importance are associated with better performance, whereas overly large bubbles correspond to cases where the model over-relies on a few dominant but non-transferable features, reducing generalization.

These observations highlight that the reduced performance of direct transfer primarily stems from mismatches between dominant feature distributions across catchments. As KL divergence increases, the kappa score declines, confirming that dissimilar environmental characteristics distort the learned feature–response relationships, regardless of the model used. Larger bubbles at high divergence values further indicate that over-dependence on a few dominant but non-transferable features amplifies this decline. Hence, direct transfer is less reliable when the physical and statistical similarity between source and target catchments is weak, limiting model generalization. These results collectively emphasize that increasing divergence in dominant environmental features constrains model transferability, underscoring the need for adaptation-based transfer learning to improve cross-catchment generalization.

Collectively, these findings highlight that the success of transfer learning for RWH site prediction depends on the degree of environmental and statistical similarity between catchments. When dominant features exhibit low divergence, models preserve stable feature–response relationships, enabling effective transfer even under limited target data conditions. Conversely, large inter-catchment divergence disrupts the stability, resulting in poor generalization during direct transfer. These insights reinforce the necessity of adaptation-based strategies that recalibrate feature relevance between domains, thereby enhancing transfer robustness and supporting reliable RWH suitability mapping in data-scarce regions.

## Conclusion

This study presents a novel TL framework for improving RWH site suitability mapping across hydro-geomorphically distinct and data-scarce catchments. By integrating the GFS derived from KL divergence, the framework systematically evaluates the feasibility of transferring models between source and target domains, providing a structured approach for cross-catchment model application.

The results demonstrate that TL performance strongly depends on the similarity between source and target catchments. When key environmental features are closely aligned, TL produces accurate and stable predictions, reflected in high kappa values and consistent model agreement. Incorporating a limited number of target-domain samples further enhanced F1-scores by 25–40% in OD, 20–30% in MH, and 35–45% in TN compared to direct transfer. Multi-source combined models outperformed single-source transfers, achieving F1-scores of 0.84 (OD), 0.82 (MH), and 0.87 (TN), respectively, highlighting the value of leveraging diverse datasets. The study highlights that transferability is governed by both the stability of influential thematic layers and the degree of divergence between source and target domains.

Overall, the proposed framework provides a scalable, transferable, and practical decision-support tool for identifying RWH sites in data-scarce regions. By enabling efficient planning of water conservation structures, it can support practitioners and government agencies, such as the Ministry of Jal Shakti and the Central Water Commission, in prioritizing water resource interventions, improving climate resilience, and contributing to long-term water security and environmental sustainability.

## Limitations and future scope

The GFS relies on thematic layers derived from heterogeneous datasets such as soil, geomorphology, and LULC, which may not fully capture fine-scale on-ground variability. Such limitations can influence similarity estimates and affect model transferability. Additionally, the framework has been tested only on three catchments; further validation is needed to ensure broader applicability across diverse regions.

Future research should integrate higher-resolution and dynamically updated datasets to better capture on-ground variability and consider climate change-informed rainfall scenarios, including non-stationary IFD curves. The framework can be applied under future climate scenarios by recalibrating the GR4J model using projected rainfall and PET inputs, allowing the ML/TL framework to reassess RWH site suitability under changing conditions, although uncertainties in climate projections may affect results. Also, expanding to additional catchments will help develop a generalized, ‘India-wide TL-based RWH model’. This foundation allows a continually evolving TL framework that updates feature weights as new data become available. We recommend leveraging multi-source datasets, systematically evaluating source–target similarity, and coupling TL with process-based hydrological models to enhance prediction reliability and interpretability.

## Supplementary Information

Supplementary Information.

## Data Availability

The supporting codes and workflows are available for peer review through a private Fig share repository: https://figshare.com/s/c82e667f369911b6dc34.
