# Supplementary material for: Transfer learning for identifying rainwater harvesting sites in training data-scarce catchments
Source: Sci Rep. 2026 May 5;16:20651. doi: 10.1038/s41598-026-51218-2 (PMC13334023; doi:10.1038/s41598-026-51218-2)
Supplement: Supplementary file 1 — Supplementary Information. [file 41598_2026_51218_MOESM1_ESM.docx]

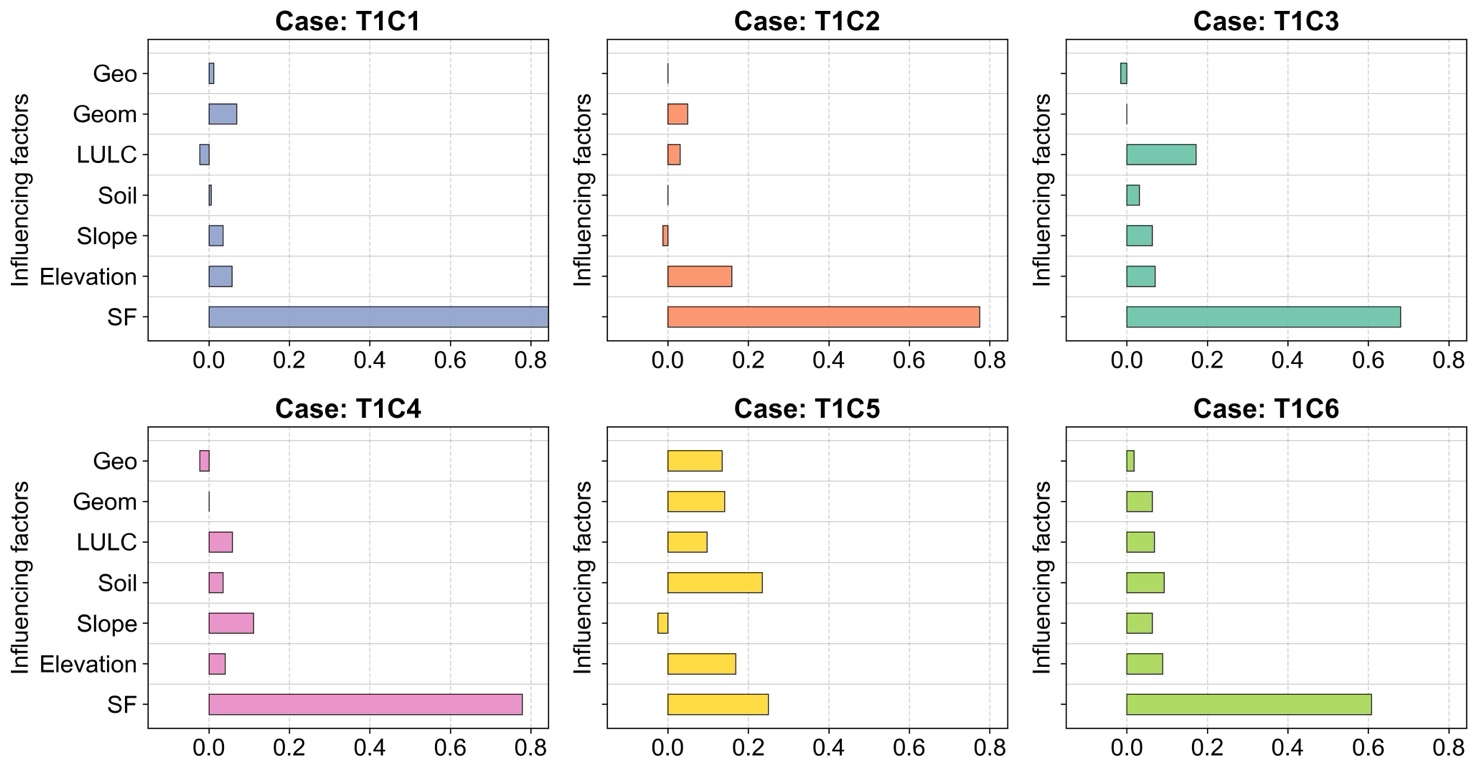


**Figure S1 : Feature importance of SVM model across cases for OD catchment (E1)**


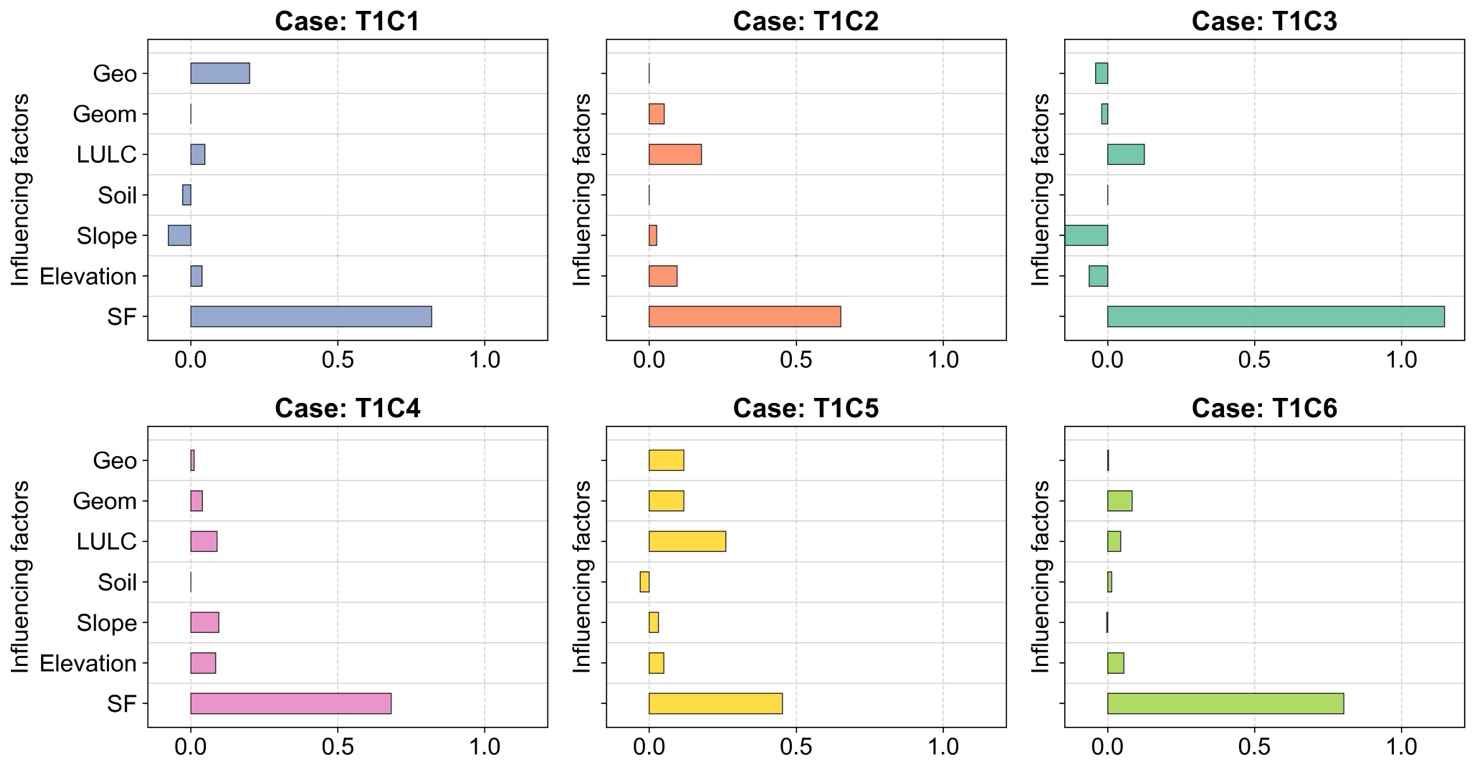


**Figure S2 : Feature importance of KNN model across cases for OD catchment (E1)**


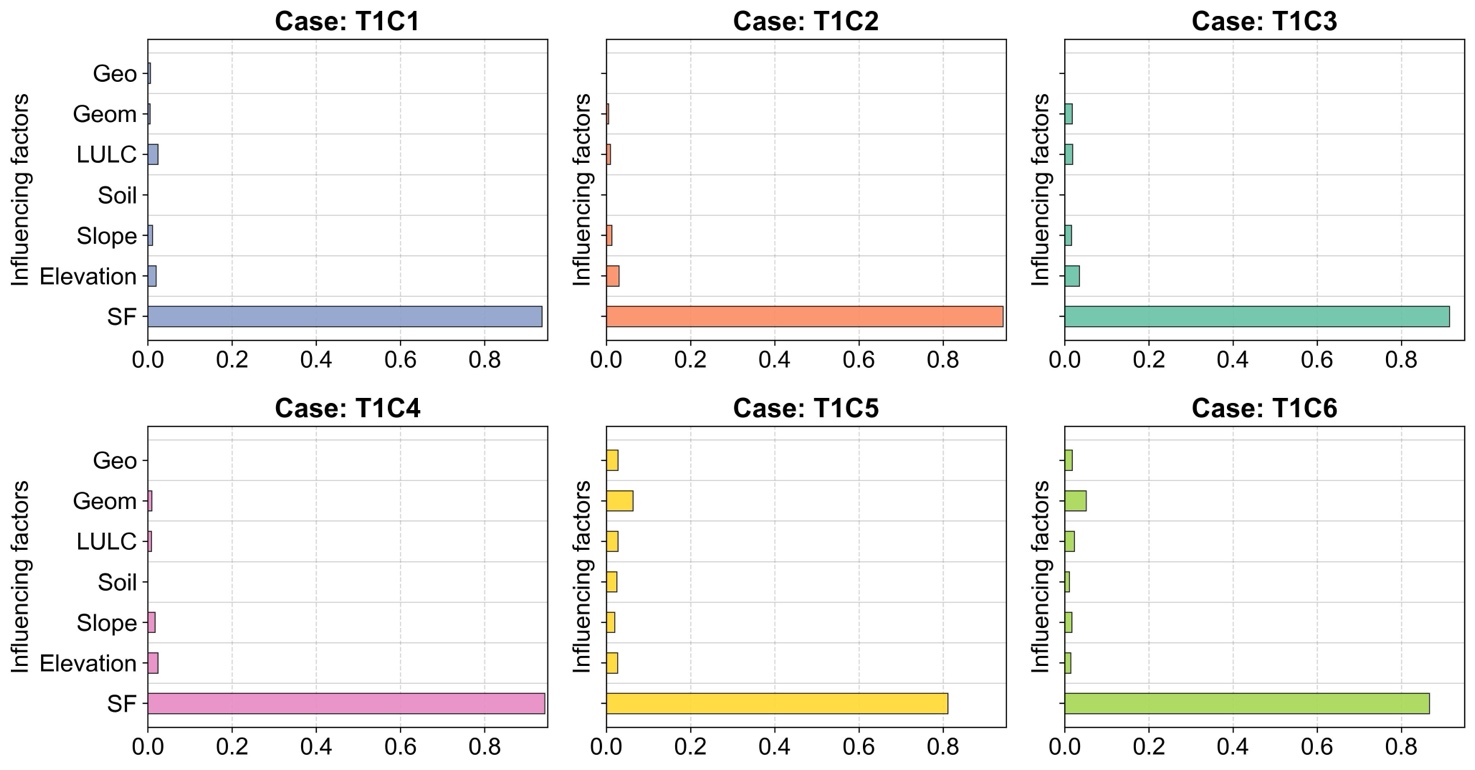


**Figure S3 : Feature importance of XGB model across cases for OD catchment (E1)**


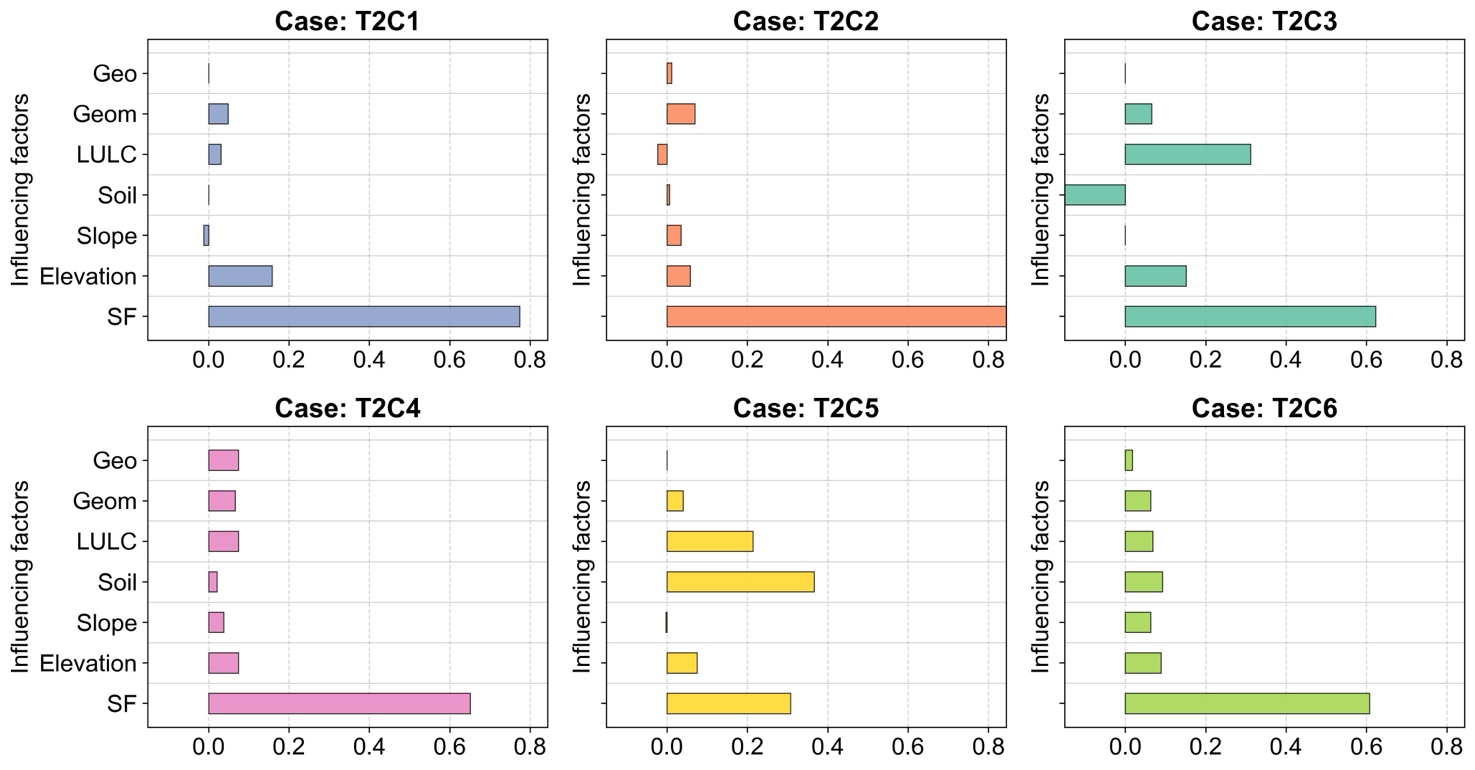


**Figure S4 : Feature importance of SVM model across cases for MH catchment (E2)**


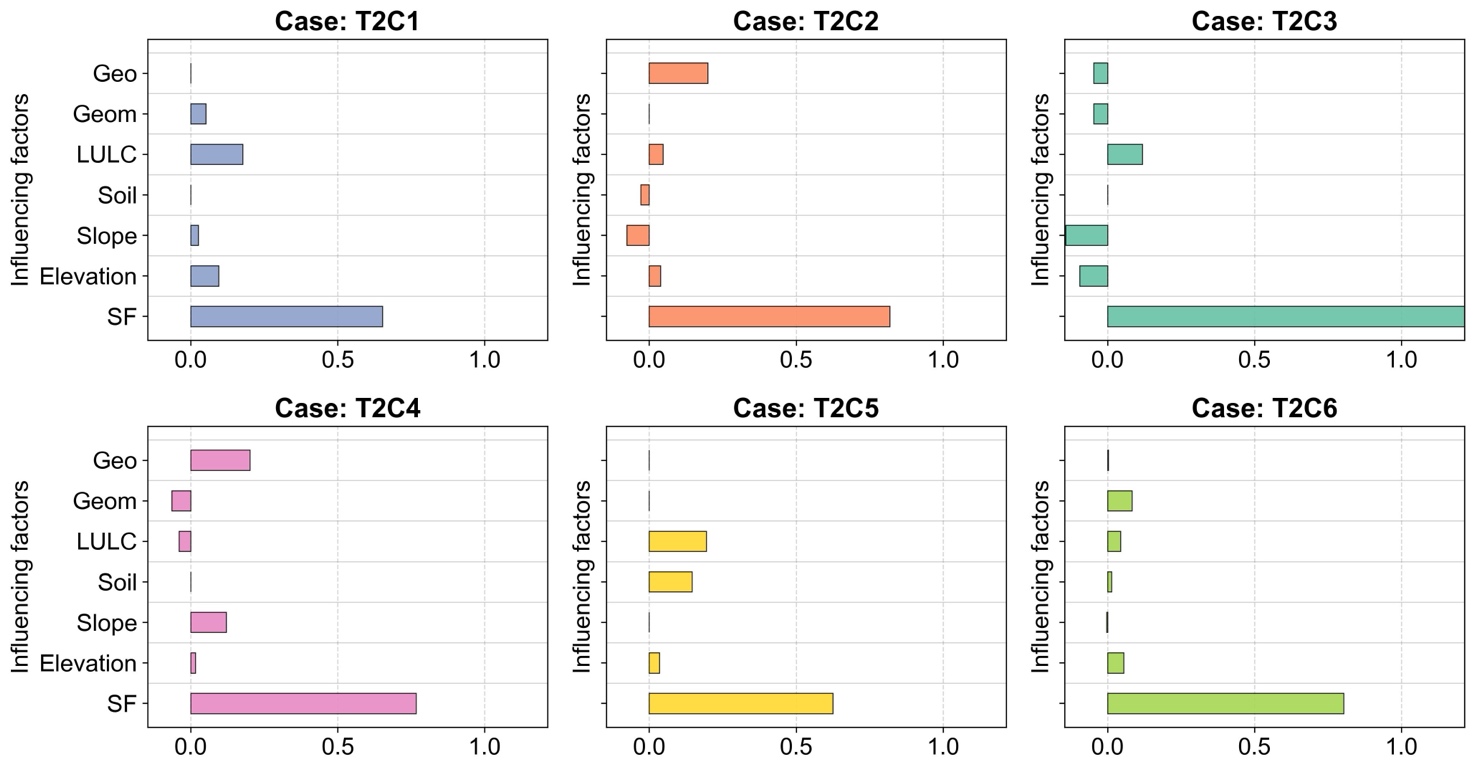


**Figure S5 : : Feature importance of KNN model across cases for MH catchment (E2)**


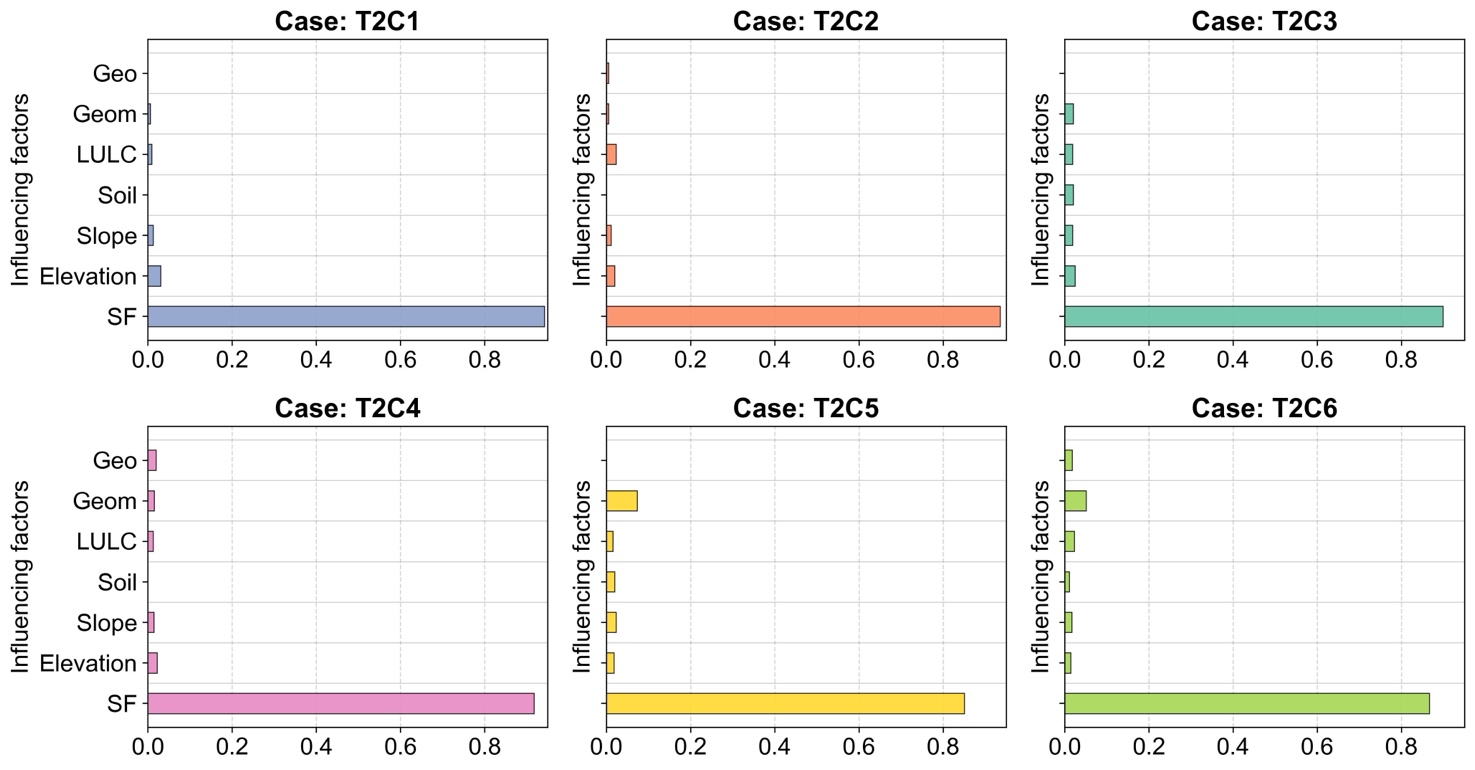


**Figure 6 : Feature importance of XGB model across cases for MH catchment (E2)**


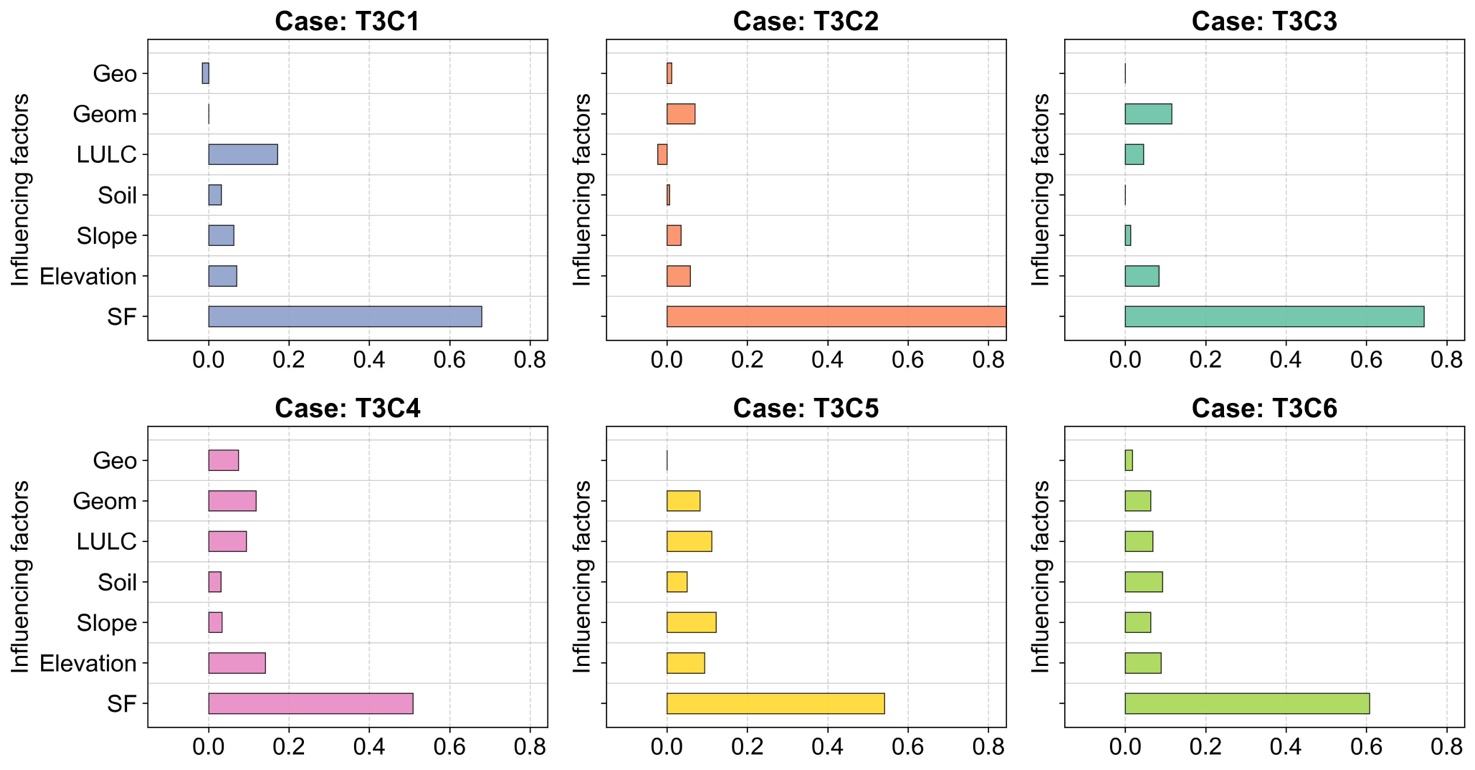


**Figure S7 : Feature importance of SVM model across cases for TN catchment (E3)**


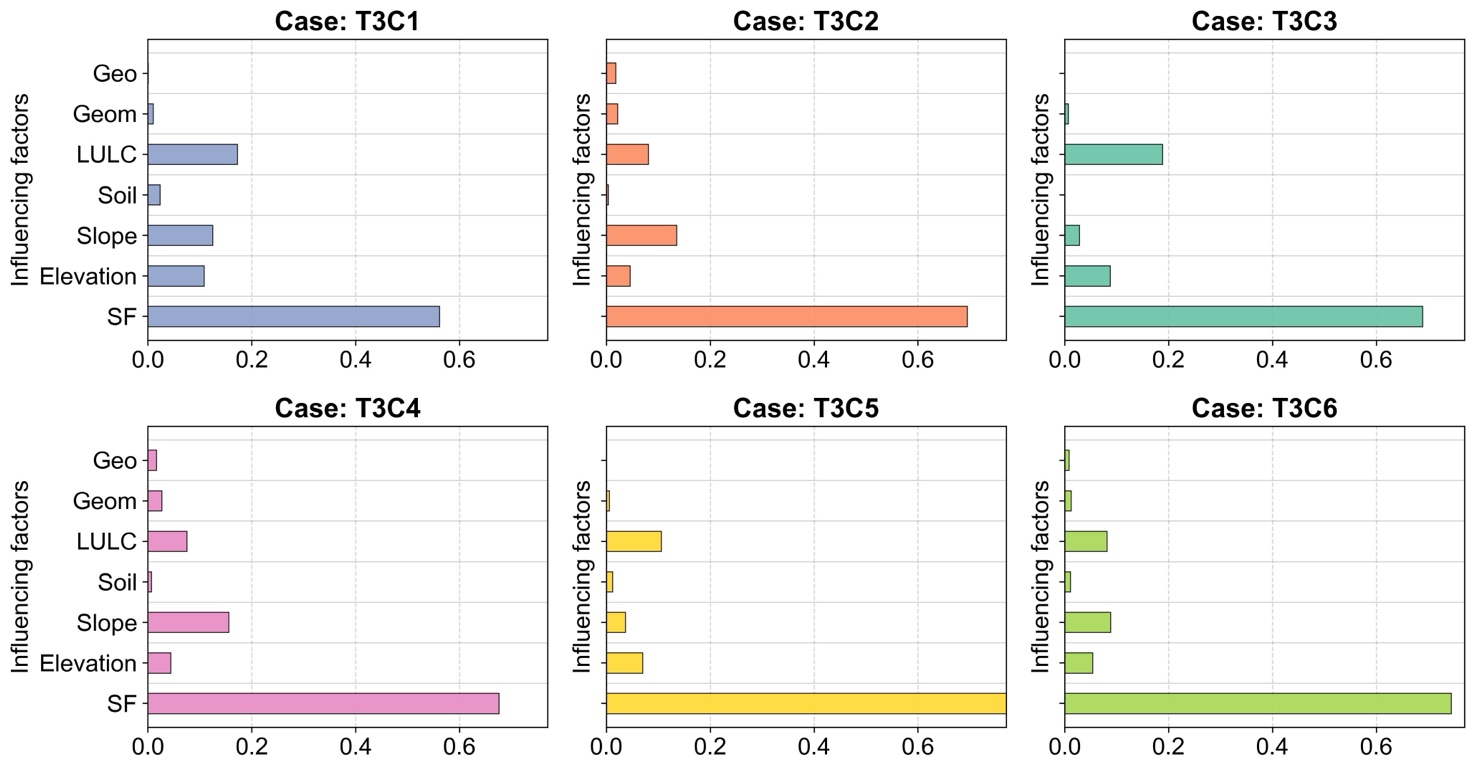


**Figure S8 : Feature importance of KNN model across cases for TN catchment (E3)**


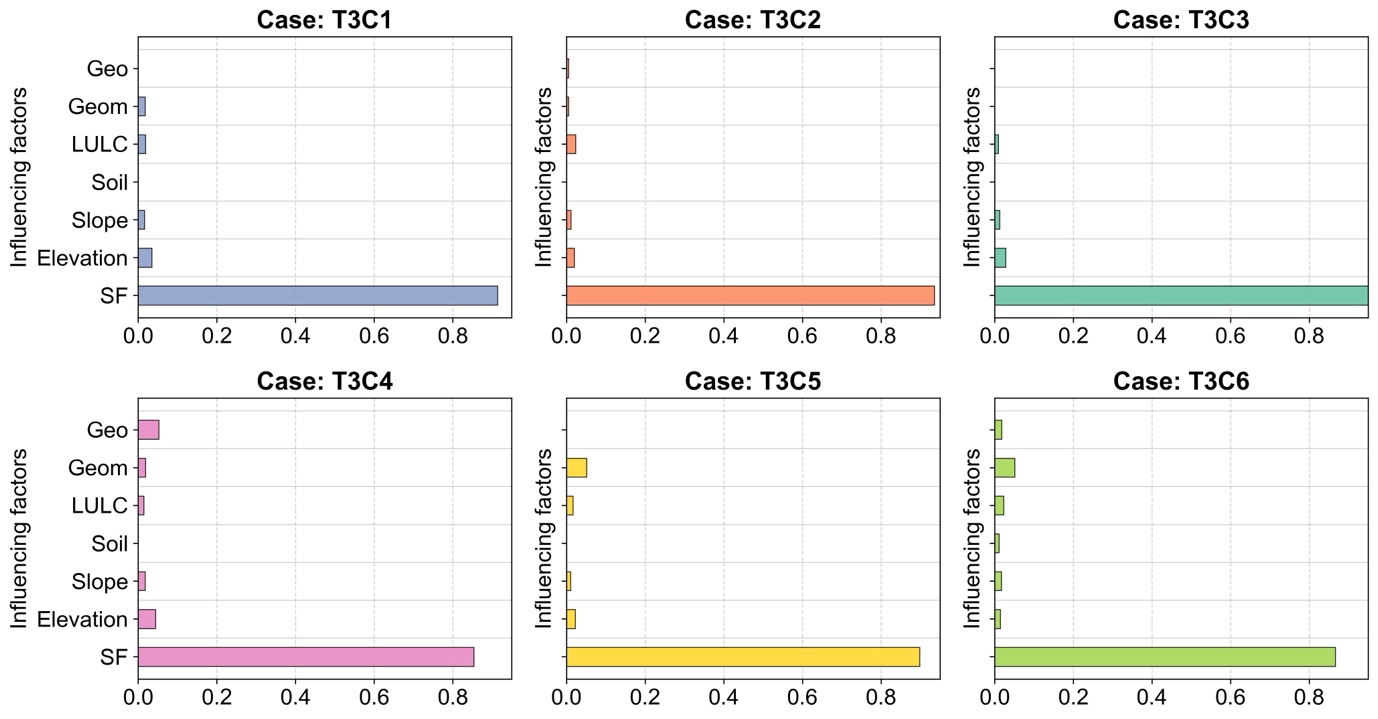


**Figure S9 : Feature importance of KNN model across cases for TN catchment (E3)**
